# Dysregulation of FGFR1 signaling in the hippocampus facilitates depressive disorder

**DOI:** 10.1038/s12276-025-01519-9

**Published:** 2025-08-15

**Authors:** Jongpil Shin, Hyeonsik Oh, Ji Hye Park, Hyunsik Bae, Chan Mo Yang, Minju Lee, Seokhwi Kim, Won Do Heo

**Affiliations:** 1 Department of Biological Sciences, Korea Advanced Institute of Science and Technology, Daejeon, Republic of Korea; 2 Forensic Medicine Investigation Division, Seoul Institute, National Forensic Service, Seoul, Republic of Korea; 3 Department of Pathology, Severance Hospital, Yonsei University College of Medicine, Seoul, Republic of Korea; 4 Department of Pathology and Translational Genomics, Samsung Medical Center, Sungkyunkwan University School of Medicine, Seoul, Republic of Korea; 5 Department of Biomedical Science, Graduate School of Ajou University, Suwon, Republic of Korea; 6 Division of Forensic Medical Examination, Department of Forensic Medicine, National Forensic Service, Wonju, Republic of Korea; 7 Department of Pathology, Ajou University School of Medicine, Suwon, Republic of Korea; 8 Korea Advanced Institute of Science and Technology Institute for the BioCentury (KIB), Daejeon, Republic of Korea; 9 Department of Brain and Cognitive Sciences, Korea Advanced Institute of Science and Technology (KAIST), Daejeon, Republic of Korea

**Keywords:** Depression, Neurological disorders, Growth factor signalling

## Abstract

Major depressive disorder (MDD) is a complex psychological disorder with a sophisticated molecular etiology. Although its connection with fibroblast growth factor receptor 1 (FGFR1) in the hippocampus is known, the precise mechanisms underlying its pathophysiology remain unclear. Here we conduct a comprehensive analysis of the molecular profile of the hippocampus in patients with MDD. We identified a distinct overexpression of FGFR1 specifically within the dentate gyrus of patients with MDD. Through the use of optogenetic techniques for the in vivo spatiotemporal dissection of FGFR1 signaling, we uncovered a sequential FGFR1–Notch–brain-derived neurotrophic factor (BDNF) pathway within the dentate gyrus, which can ultimately induce adult hippocampal neurogenesis, significantly contributing to antidepressant effects. We discovered that the dysregulation of this axis by the protein Numb, which demonstrates an age-related increase in individuals with MDD, is closely associated with the development of depressive phenotypes. Remarkably, targeting Numb to restore this axis effectively reversed the depressive phenotype, thus offering new insights into potential therapeutic strategies.

## Restoring numb function reverses depression in patient with MDD

Major depressive disorder (MDD) is a mental health condition that affects mood and thinking. This study examines the role of a protein called FGFR1 in the brain’s hippocampus, a region linked to mood regulation. Researchers used advanced techniques to study brain tissue from patients with MDD and mice. They found that FGFR1 is overexpressed in the hippocampus of patients with MDD. Using optogenetics (a method to control the signaling pathway of cells with light), they discovered a new pathway involving FGFR1, Notch and brain-derived neurotrophic factor (BDNF) proteins that promotes neurogenesis and reduces depression symptoms. The study shows that activating this pathway can help alleviate depression, especially in younger individuals. However, in older adults, a protein called Numb disrupts this process. By targeting Numb, researchers hope to develop new treatments for depression, particularly in older adults.

This summary was initially drafted using artificial intelligence, then revised and fact-checked by the author.

## Introduction

Major depressive disorder (MDD) is a complex psychological disease marked by disturbances in mood and cognition^1–3^, affecting individuals across various age groups^4^. The intricate molecular basis of depression involves a complex interplay of genetic, neurobiological and environmental factors^3^, rendering its treatment challenging^5,6^. Beyond the traditional focus on neurotransmitter imbalances, particularly serotonin, norepinephrine and dopamine^1^, recent studies have shifted toward the understanding of the role of the hypothalamic–pituitary–adrenal (HPA) axis and neurotrophic factors, a theory that depression is linked to a deficiency or dysregulation of neurotropic factors, in MDD’s pathology^3^.

Elevated cortisol levels in patients with MDD^7,8^ and glucocorticoid-induced depressive-like behavior in rodents highlight the important role of the HPA axis in depression^9,10^. The hippocampus, known for its role in providing inhibitory feedback on glucocorticoid release and its vulnerability to structural changes under chronic stress, is a critical area implicated in MDD^11–14^. Neurotrophic factors, which are proteins that support neuron growth, survival and maintenance, play a vital role in the brain function and HPA axis regulation. These factors have been linked to hippocampal atrophy in MDD^15–17^, with reduced levels of brain-derived neurotrophic factor (BDNF) and fibroblast growth factor 2 (FGF2), along with an increase in fibroblast growth factor receptor 1 (FGFR1), observed in the hippocampal tissue of patients with MDD^16,18^. Moreover, antidepressant treatment have been shown to mitigate depressive effects by upregulating FGF2 in both human^19,20^ and rodent models^21–24^. Despite these insights, current literature primarily addresses the relationship between these molecular components and depressive symptoms, without fully elucidating mechanistic details.

The advent of optogenetic techniques, which permit precise spatiotemporal activation of signaling pathway components beyond what is possible with ligand treatments or genetic manipulation^25–27^. It has proven instrumental in dissecting the complex dynamics of cellular signaling pathways and their dysfunctions in diseases^26^.

In this article, we highlight a specific overexpression of FGFR1 in the dentate gyrus of the MDD hippocampus through comprehensive transcriptomic profiling on postmortem brain tissue. Employing optogenetic techniques^26,28,29^, we uncovered a novel FGFR1–Notch–BDNF axis that plays a crucial role in inducing adult hippocampal neurogenesis (AHN) and mediating antidepressant effects. We found that the dysregulation of this axis due to increased expression of Numb with age, contributes to the depressive-like phenotype. Our findings also demonstrate that inhibiting Numb to restore this axis yields antidepressive effects, offering new potential therapeutic implications.

## Materials and Methods

### Human subjects research

Postmortem brain tissue from six patients with MDD and nine non-MDD individuals was collected between July 2020 and June 2021 at the National Forensic Service Seoul Institute. Five patients with MDD were confirmed via postmortem blood screening for antidepressant medications, and one was confirmed based on medical history and family reports. Clinical characteristics, medical history and cause/mode of death are detailed in Supplementary Tables 1 and 2. Informed consents from the human subjects were waived following the policy of the Institutional Review Board of the National Forensic Service. The study protocol was approved by the Institutional Review Board of the National Forensic Service (906-210715-BR-001-01).

The hippocampal tissue was obtained from postmortem brains and promptly fixed in 10% neutral formalin (Biosesang) for 24 h, after which a paraffin block was created. The formalin-fixed, paraffin-embedded tissue was then sliced to generate ten slides of 10-μm-thick sections for RNA isolation in sequencing studies and 4-μm-thick sections for immunohistochemical (IHC) staining. The hippocampal subregions (dentate gyrus, CA3, CA2 and CA1) were meticulously dissected from tissue section slides under high-magnification light microscopy by three board-certified pathologists (H.B., M.L. and S.K.), each with over 5 years of experience in neuropathology. The dissection was performed using a sharp blade, with reference to the human brain atlas (https://atlas.brain-map.org/) to ensure precision and accuracy.

### Animals

C57BL/6J inbred mice, homozygous *Fgfr1*^*flox/flox*^ strain (B6.129S4-Fgfr1tm5.1Sor/J) and CamkIIα-cre strain (B6.Cg-Tg(Camk2a-cre)T29-1Stl/J) were obtained from Jackson Laboratory and bred in the KAIST animal facility. The mice were subjected to stereotactic surgery at the age of 7 or 8 weeks or 1 year, depending on the behavior paradigm. All mice were given free access to food and water. All experimental procedures were performed in accordance with the guidelines of the Institutional Animal Care and Use Committee at KAIST.

### DNA construction

To construct optoFGFR1-HA, the PHR(E281A) mutant domain of cryptochrome 2 with an HA-tag was amplified by polymerase chain reaction (PCR) from optoFas^26^ and inserted into The CMV::lyn-cytFGFR1-Cry2PHR-mCitrine construct^27^ using restriction enzymes BamHI and NotI. The following primers were used F:5′-GTAGGATCCCATGAAGATGGACAAAAAGACCA-3′, R: 5′-GCGGCCGCGGCGCGCCTTAAGCATAATCTGGAACATCATATGGATATACCGGTGGGCTGCCGCTGCCGGCAGCACCGATCATAATCTGC-3′. AAV-hSyn-DIO-optoFGFR1-HA was constructed by Gibson Assembly Cloning, which was used to replace cytTrkB–EGFP^29^ with PCR-amplified optoFGFR1-HA in the pAAV-hSyn-DIO-optoTrkB-EGFP vector. The following primers were used F:5′- ATAGGATACTTTATACGAAGTTATGGCCACCATGGGATGTATAAAATCAAAAGG-3′, R: 5′- GCATACATTATACGAAGTTATGGGCGGCCGCGGCGC-3′. AAV-EF1α-DIO-DSE-mCherry-PSE-shRNA-Scramble was constructed by restriction enzyme cloning with annealed oligos. AvrII and EcoRI were used to digest pDIO-DSE-mCherry-PSE-MCS (Addgene cat. no. 129669). The following primers were used F:5′-CTAGGTTCTCCGAACGTGTCACGTCCTGA-3′, R:5′- TGGGTCAGGACGTGACACGTTCGGAGAAc-3′, F:5′-CCCAACGTGACACGTTCGGAGAATTTTTG-3′, R:5′- TGGGTCAGGACGTGACACGTTCGGAGAAc-3′. AAV-EF1α-DIO-DSE-mCherry-PSE-shRNA-Numb was constructed by restriction enzyme cloning with annealed oligos. AvrII and EcoRI were used to digest AAV-EF1α-DIO-DSE-mCherry-PSE-shRNA-Scramble and oligo duplex was purchased from Origene (cat. no. SR417915). pAAV-hSyn-DIO-EGFP vector was purchased from Addgene (cat. no. 50457). Lenti-CMV-TurboGFP-shRNA-Notch1 was purchased from Origene (cat. no. TL704320).

### In vivo pharmacological treatment

The corticosterone administration protocol followed the methodology described by Zhao et al.^9^. Male C57BL/6J and *Fgfr1*^*flox/flox*^ (B6.129S4-Fgfr1tm5.1Sor/J) mice were housed under a 12-h light–dark cycle (lights off at 20:00, 25 °C ± 1 °C) with ad libitum food and water. Mice were randomly assigned to three groups: (1) corticosterone (20 mg kg^−1^, Sigma, cat. no. 27840) subcutaneously in 0.1% DMSO/Tween-80 saline (5 ml kg^−1^) for 21 days, (2) vehicle for 21 days or (3) vehicle for 14 days followed by corticosterone for 7 days. Depressive-like behaviors were assessed via the sucrose preference, open field and tail suspension tests before biochemical analysis, including western blot and quantitative real-time PCR (qRT–PCR).

The 5-ethynyl-2′-deoxyuridine (EdU) administration protocol followed the methodology of Hong et al.^30^. A 10 mg ml^−1^ EdU (Invitrogen, cat. no. A10044) solution was prepared in saline and injected intraperitoneally (100 mg kg^−1^). In CamkIIα-cre mice (B6.Cg-Tg(Camk2a-cre)T29-1Stl/J), EdU was administered 12 h before blue light stimulation. In *Fgfr1*^*flox/flox*^ mice, EdU was administered before corticosterone treatment, followed by 7 days of vehicle or corticosterone. Depressive-like behaviors were assessed before biochemical analysis.

The ANA-12 administration schedule followed the methodology of Kim et al.^26^. A stock solution was prepared in 20 nM DMSO (Merck, cat. no. 317275-500 mL) and diluted in saline. Male CamkIIα-cre mice received 0.5 mg kg^−1^ intraperitoneally 8 h after blue light stimulation.

*N*-[*N*-(3,5-difluorophenacetyl)-l-alanyl]-*S*-phenylglycine *t*-butyl ester (DAPT) (Selleckchem, cat. no. S2215) was similarly prepared and administered intraperitoneally (100 mg kg^−1^) 12 h before blue light stimulation in male CamkIIα-cre mice.

### CUMS model construction

C57BL/6J inbred mice and homozygous *Fgfr1*^*flox/flox*^ (B6.129S4-Fgfr1tm5.1Sor/J) mice were subjected to ten different mild stressors: (1) food and water deprivation (24 h), (2) 45° cage tilt (8 h), (3) light cycle reversal (24 h), (4) forced swimming (25 °C ± 1 °C, 5 min), (5) Tail pinching (1 cm from the end, 5 min), (6) moist bedding (8 h), (7) no stress (24 h), (8) cold exposure (10 °C, 30 min), (9) sleep deprivation (12 h) and (10) spatial isolation (1 h).

These stressors were randomly scheduled over 28 days, with one to two stressors applied daily in an unpredictable combination. Control mice were individually housed for the same duration. Behavioral experiments—open field test, tail suspension test and sucrose preference test—were conducted weekly on a fixed day before, during and after the chronic unpredictable mild stress (CUMS) paradigm to assess depressive-like behaviors. Body weight was recorded the day before each behavioral assessment.

### Behavior experiments

The sucrose preference test (SPT) was conducted in a single cage with two bottles containing either water or 0.5% sucrose solution (Sigma, cat. no. S7903). Bottle weights were recorded before and after 24-h exposure. The sucrose preference index was calculated as sucrose consumption over total fluid intake.

The open field test assessed locomotion and anxiety-related factors. Mice were placed in a 40 cm × 40 cm × 40 cm acrylic chamber and allowed to explore freely for 30 min. Using Ethovision XT (Noldus), total distance, average speed and time in the central 20 cm × 20 cm area were analyzed. The anxiety index was calculated as {(total time) − (time spent in the central area)}/(time spent in the central area).

Following a 24-h rest, the tail suspension test was performed. Mice were suspended 60 cm above the ground using sticky tape placed ~1 cm from the tail tip, with a plastic straw inserted at the base. Immobility time was recorded during the last 6 min of a 7-min trial, with immobility defined as passive, motionless hanging.

### Western blot analysis

Proteins were extracted from frozen tissue using lysis buffer (Intron, cat. no. 17081) per the manufacturer’s protocol, then boiled with sample buffer (Biosesang). The samples were loaded onto 4–12% Bis–Tris Plus Gels, separated via SDS–polyacrylamide gel electrophoresis and transferred to nitrocellulose membranes (iBlot; ThermoFisher) using iBlot Transfer Stack. The membranes were blocked with TBS/Odyssey blocking buffer, incubated overnight at 4 °C with primary antibodies (dilutions below), washed with TBS-T (0.1% Tween-20, Sigma, cat. no. P2287) and incubated with IR-Dye-conjugated secondary antibodies (Li-COR). Imaging was performed using the LI-COR Odyssey Clx system, and the data were analyzed with Image Studio software. The following antibodies with diluent factors were used in this study: rabbit anti-FGFR1 (D8E4) (Cell Signaling Technology, cat. no. 9740) 1:1,000, rabbit anti-Cre (Abcam, cat. no. ab190177) 1:1,000, rabbit anti-Notch1 (D1E11) (Cell Signaling Technology, cat. no. 3608) 1:1,000, rabbit anti-Hes1(D6P2U) (Cell Signaling Technology, cat. no. 11988) 1:1,000, rabbit anti-FGF2 (E5Y6M) (Cell Signaling Technology, cat. no. 46879) 1:1,000, rabbit anti-Numb (Cell Signaling Technology, cat. no. 2756) 1:1,000, Rabbit anti-BDNF (EPR1292) (Abcam, cat. no. ab108319) 1:1,000, mouse anti-GAPDH (GA1R) (ThermoFisher, cat. no. MA5-15738) 1:3,000.

### Quantitative real-time polymerase chain reaction (qRT–PCR)

Hippocampal tissue was collected on ice immediately after light stimulation or behavioral tests in a dark room, snap-frozen in liquid nitrogen and stored at −80 °C until RNA isolation. The primary neurons were cultured in 12-well plates (CellVis, cat. no. P12-1.5H-N) and treated with a viral solution Multiplicity of infection (MOI of 8000). Following light stimulation or basic FGF (PeproTech, cat. no. 400-29) treatment, neurons were collected, centrifuged and stored at −80 °C for RNA isolation. RNA was extracted using the PureLink Mini Kit (Ambion, cat. no. 12183018A) per the manufacturer’s protocol. cDNA synthesis was performed with 200 ng RNA using the SuperScript III First-Strand Synthesis System (Invitrogen, cat. no. 18080-051). qRT–PCR was conducted on a CFX Opus 96 Real-Time PCR System (Bio-Rad) using the Solg 2× Real-Time PCR Smart kit and Evagreen dye (Solgent, cat. no. SRH72-M40h) with a 50-cycle, three-step protocol. The following primers were used: rat GAPDH, F:5′-ACAAGATGGTGAAGGTCGGTGTGA-3′, R:5′-AGCTTCCCATTCTCAGCCTTGACT-3′; rat SDC4, F:5′-AAGTGCTGACAGCTCATGCT-3′, R:5’-GAAAGA ACATCTGTGCATCCC-3′; rat Hes1F:5′-CGACACCGGACAAACCAAA-3′, R:5′-GAATGTCTGCCTTCTCCAGCTT-3′; rat Hes5 F:5′- ATGCTCAGTCCCAAGGAGAA-3′, R:5′-CTCCAGCAGCAGTTTCAGC-3′; rat FGF2 F:5′-GGCTTCTTCCTGCGCATCCA-3′, R:5′- GCTCTTAGCAGACATTGGAAGA-3′; rat BDNF F:5′-GGTTCGAGAGGTCTGACGAC-3′, R:5′-CAAAGGCACTTGACTGCTGA-3′; rat Hey1 F:5′-CGACGAGACCGAATCAATAAC-3′, R:5′-CAAACTCCGATAGTCCATAGCC-3′; rat Hey2 F:5′-TGACAGAAGTGGCGAGGTA-3′, R:5′-CACAGGTGCTGAGATGAGAG-3′; rat Rest F:5′-ACTCGACACATGCGTACTCACTCA-3′, R:5′-CTTGCGTGTCGGGTCACTTC-3′; rat Runx1 F:5′-AACCCTCAGCCTCAAAGTCA-3′, R:5′-GGGTGCACAGAAGAGGTGAT-3′; rat Notch1 F:5′-TGAATGGAGGGAGGTGCGAAGTGG-3′, R:5′-GCGGGCACCGGCACTTGTATT-3′; mouse GAPDH F:5′-CTGAGTATGTCGTGGAGTCTACTGG-3′, R:5′-GTCATATTTCTCGTGGTTCACACC-3′; mouse FGFR1 F:5′-AACCTCTAACCGCAGAAC-3′, R:5′-GAGACTCCACTTCCACAG-3′.

### Immunohistochemistry

Mice brains were fixed in 4% paraformaldehyde (EMS, cat. no. 15713) in DPBS at 4 °C for 24 h, then sectioned into 45-μm coronal slices using a VT1200S vibratome (Leica). The slices were mounted with DAPI (Vector Laboratories, cat. no. H-1200). The mice were perfused with PBS before fixation in 4% paraformaldehyde. The slices were blocked with 5% normal goat serum (Abcam, cat. no. ab7481) including 0.3% Triton-X (Sigma, cat. no. X100) in DPBS for 1 h, washed and incubated overnight at 4 °C with primary antibodies, including anti-HA-Tag rabbit for HA-tagged OptoFGFR1. After washing, the slices were incubated with secondary antibodies for 1 h at room temperature, washed and imaged with a confocal microscope. The following primary antibodies were used: rabbit anti-phospho-p44/42 MAKR(Erk1/2) (Thr202/Tyr204) (Cell Signaling Technology, cat. no. 4370) 1:10,000; rabbit anti-phospho-S6 Ribosomal Protein (S235/S236) (Cell Signaling Technology, cat. no. 4858) 1:400; rabbit anti HA-tag (C29F4) (Cell Signaling Technology, cat. no. 3724) 1:1,000; rabbit anti-BDNF (Alomone, cat. no. ANT-010) 1:300; mouse anti-Ha-tag (6E2) (Cell Signaling Technology, cat. no. 2367) 1:1,000, rabbit anti-TBR2 (abcam, cat. no. ab183991) 1:1,000, rabbit anti-Ki-67 (abcam, cat. no. ab15580) 1:500.

IHC staining of human tissue was performed with primary antibodies and 4-μm-thick tissue sections of formalin-fixed, paraffin-embedded tissues using a BenchMark XT automated immunostainer (Ventana Medical Systems). The following primary antibodies were used: rabbit anti-FGFR1 (D8E4) (Cell Signaling Technology, cat. no. 9740, 1:400), rabbit anti-BDNF (Invitrogen, cat. no. PA5-85730, 1:100) and mouse anti-Numb (Origene, cat. no. CF502168, 1:150). The *H*-score for quantitative evaluation of IHC staining was calculated as follows

$${{H}}\text{-}{\rm{score}}=(3\times\,( \% {\rm{proportion}}\; {\rm{showing}} 3+{\rm{intensity}}\; {\rm{expression}}))+(2\times( \% {\rm{proportion}}\; {\rm{showing}} 2+{\rm{intensity}}\; {\rm{expression}}))+(1\times( \% {\rm{proportion}}\; {\rm{showing}} 1+{\rm{intensity}}\; {\rm{expression}})).$$

### RNA sequencing data analysis

QuantSeq 3′ mRNA-Seq reads were aligned using Bowtie2 (Langmead and Salzberg, 2012) with indices generated from genome assembly or representative transcript sequences. Alignment files were used for transcript assembly, abundance estimation and differential gene expression analysis. Differentially expressed genes (DEGs) were identified using Bedtools (Quinlan, 2010) based on unique and multiple alignments. Read count data were normalized using the trimmed mean of M-values (TMM) + counts per million (CPM) method in EdgeR (Bioconductor, R Development Core Team, 2020). Gene classification was performed using DAVID and Medline databases.

### Statistical analysis

The statistical analysis was performed using GraphPad Prism 7.00 (GraphPad Software). The data are presented as the mean ± s.e.m. For two-group comparisons, *t*-tests (unpaired) were used, with the Mann–Whitney test for non-normally distributed data. For multiple groups, one-way analysis of varaince (ANOVA) with Tukey’s post hoc test was used, and for two independent variables, a two-way ANOVA with Šídák’s post hoc test was applied.

The statistical analytic results are summarized in the Supplementary Information.

Additional methodologies including primary hippocampal neuron culture, virus production, in vitro viral transduction, stereotaxic viral injection, light-emitting diode (LED) stimulation in vitro, light stimulation in vivo, mouse tissue preparation, EdU incorporation and quantification of EdU^+^ cells, RNA isolation, library preparation and sequencing, gene set enrichment analysis (GSEA) and conventional animal facility can be identified in the Supplementary Information.

## Results

### Dentate gyrus specific upregulation of FGFR1 in patients with MDD

**Fig. 1:**
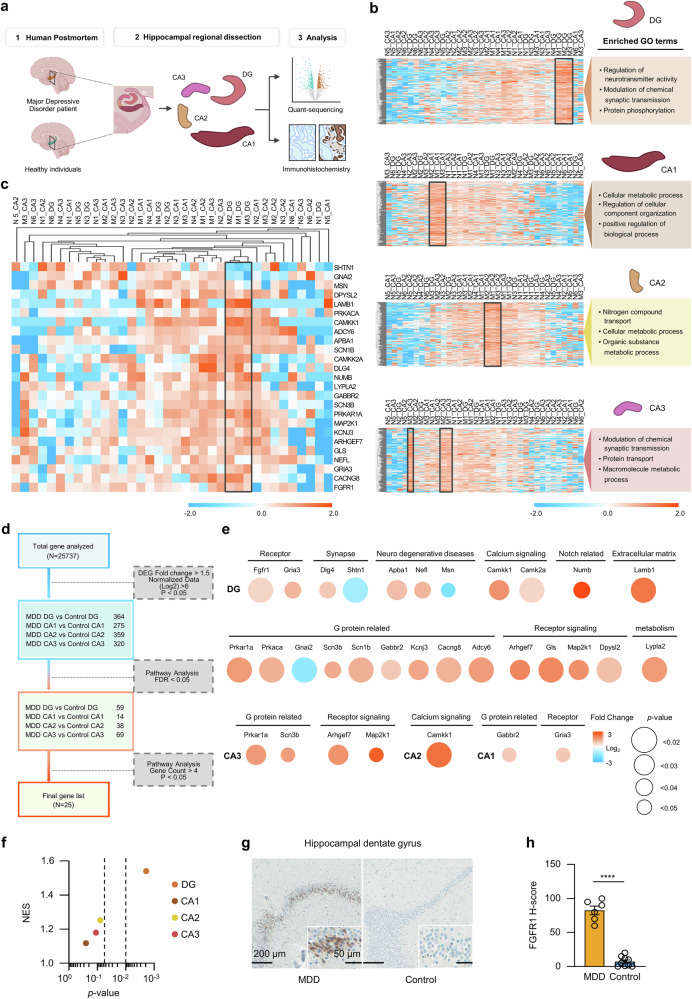
Regional transcriptome analysis in the human hippocampus identifies dentate gyrus-specific FGFR1 elevation in MDD. **a** A schematic representation of patients with MDD and control human postmortem hippocampus performing the regional dissection and analysis. **b** Heat maps showing expression levels of the top 200 genes in each subregion of the hippocampus–dentate gyrus, CA1, CA2, CA3, with enriched GO terms. **c** A heat map showing the expression levels of filtered genes observed in DAVID analysis. **d** A schematic table showing filtering process of expression levels of the genes in each region with hippocampal subregions of patients with MDD compared with the normal control. **e** A dot plot showing expression levels of the filtered genes in each region with filtered gene sets of hippocampal subregions of patients with MDD compared with the normal control. The data are represented as dot plot; samples included *n* = 6 for normal control and *n* = 3 for with MDD in each group. An unpaired two-tailed *t*-test was used for statistical analysis. **f** A dot plot showing normalized enrichment score and *P* value of patients with MDD and control dentate gyrus using a GSEA of Reactome downstream signaling of activated FGFR in the hippocampal subregions. **g** Representative images showing the expression of FGFR1 in human patients with MDD and normal control dentate gyrus. Scale bar, 200 μm (inset 50 μm). **h** A quantification of the data shown in **g**. The data are represented as means ± s.e.m.; samples included *n* = 9 for normal control and *n* = 6 for with MDD in each group. An unpaired two-tailed *t*-test was used for statistical analysis. (*t*(13) = 12.81, *P* < 0.0001). ****P < 0.0001.

To investigate the region-specific expression of genes, hippocampal tissues were collected from six patients with MDD and nine healthy individuals and were partitioned into four distinct subregions: dentate gyrus, CA3, CA2 and CA1. RNA sequencing was performed in each subregion from three MDD tissues and six healthy control tissues with sufficient RNA quality (Fig. 1a and Supplementary Table 1). The transcriptomic analysis revealed that the gene expression profile within the dissected hippocampal subregions is unique to each area (Supplementary Fig. 1). In examining the top 200 genes with the most variability within each subregion of patients with MDD, we noticed that dentate gyrus was specifically enriched for Gene Ontology (GO) terms related to ‘regulation of neurotransmitter activity’, ‘modulation of chemical synaptic transmission’ and ‘protein phosphorylation’. This suggests the heightened activation of neurons within dentate gyrus. Conversely, other regions (CA3, CA2 and CA1) were enriched in GO terms mainly related to metabolic processes (Fig. 1b). To identify specific genes that showed a significant increase in expression, we applied filters for fold change of DEGs, false discovery rate (FDR) and the gene count in the pathway analysis at a commonly used threshold. Significantly expressed genes were identified through filters: fold change >1.5, normalized log_2_ data >6 and a *P* value <0.05. DAVID pathways analysis of these genes, with a false discovery rate <0.05, revealed 25 genes enriched in more than three pathways in the MDD hippocampus. This process identified 25 upregulated genes across the four subregions of hippocampus, with a notable increase in expression predominantly observed in the dentate gyrus (Fig. 1c, d). Among these, FGFR1 was identified as the sole growth factor receptor significantly overexpressed in the dentate gyrus (Fig. 1e). Further GSEA for Reactome ‘downstream signaling of activated FGFR (M27510; R-HSA-5654687)’ reveals a specific upregulation of FGFR1 signaling cascade in the dentate gyrus compared with other hippocampal subregions (Fig. 1f and Supplementary Fig. 2a–d). IHC staining confirmed the selective overexpression of FGFR1 in the dentate gyrus of patients with MDD, consistent with the RNA sequencing analysis (Fig. 1g, h and Supplementary Fig. 3a, b). These findings highlight a distinctive upregulation of FGFR1 in the dentate gyrus of the hippocampus in patients with MDD.

### Association of increased FGFR1 expression in dentate gyrus with antidepressive effects

**Fig. 2:**
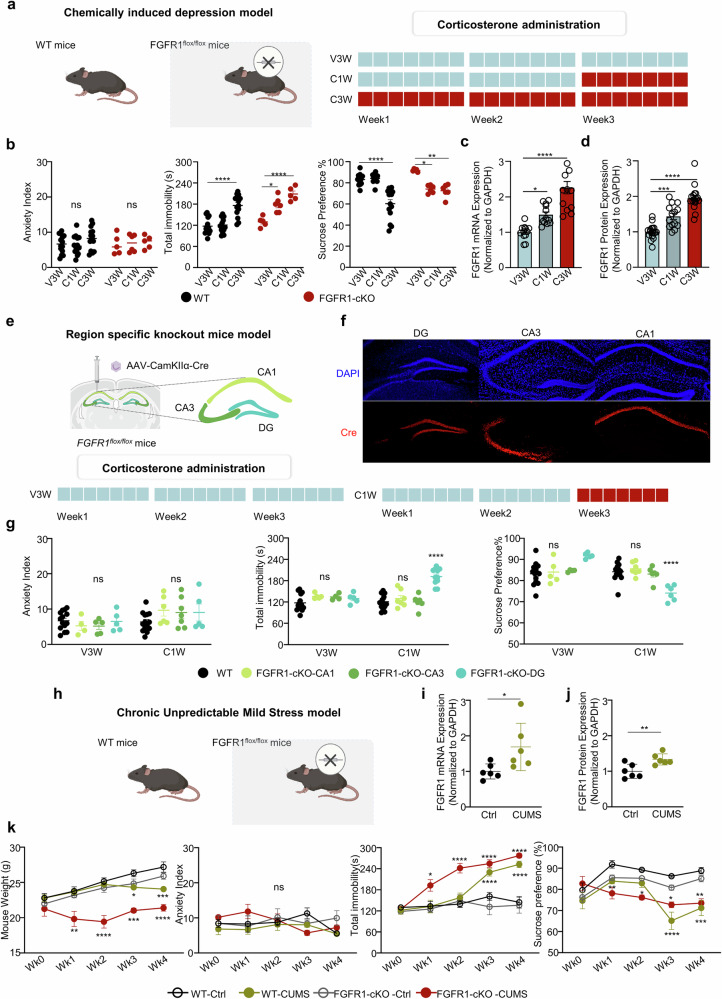
cKO of FGFR1 in the dentate gyrus accelerates depressive-like behaviors in mouse depression models. **a** A schematic representation of chemically induced mouse depression model with WT and *Fgfr1*^*flox*/*flox*^ mice. **b** Anxiety index, total immobility time in tail suspension test and sucrose preference in WT mice and *Fgfr1*^*flox*/*flox*^ mice. The experimental groups included: administration of vehicle for 3 weeks (V3W); administration of vehicle for 2 weeks, followed by corticosterone for 1 week (C1W); and administration of corticosterone for 3 weeks (C3W). WT mice (black) and FGFR1-cKO mice, in which a Cre-virus was injected into the hippocampal dentate gyrus of *Fgfr1*^*flox/flox*^ mice (red), were used. The data are presented as means ± s.e.m.; *n* = 14 WT mice, *n* = 5 FGFR1-cKO(V3W), *n* = 6 FGFR1-cKO (C1W) and *n* = 5 FGFR1-cKO (C3W) mice were included for each condition. For anxiety index, a two-way ANOVA revealed no significant main effects or interaction between genotype and corticosterone treatment (interaction: *F*(2, 52) = 0.3102, *P* = 0.7346; genotype: *F*(1, 52) = 0.0856, *P* = 0.7709; corticosterone treatment: *F*(2, 52) = 0.7861, *P* = 0.7861). Post hoc comparisons using Tukey’s multiple comparisons showed no pairwise comparisons reached statistical significance. For total immobility, a two-way ANOVA revealed significant main effects of genotype (*F*(1, 52) = 23.76, *P* < 0.0001), corticosterone treatment (*F*(2, 52) = 31.51, *P* < 0.0001) and a significant interaction between genotype and corticosterone treatment (*F*(2, 52) = 3.603, *P* = 0.0343). Post hoc comparisons using Šídák’s multiple comparisons test showed significant differences between several group pairs. (WT V3W versus WT C3W, *P* < 0.0001; FGFR1-cKO V3W versus FGFR1-cKO C1W, *P* = 0.0213; FGFR1-cKO V3W versus FGFR1-cKO C3W, *P* < 0.0001). For sucrose preference, a two-way ANOVA revealed significant main effects of corticosterone treatment (*F*(2, 53) = 26.89, *P* < 0.0001) and a significant interaction between genotype and corticosterone treatment (*F*(2, 53) = 8.859, *P* = 0.0005) but no significant main effect of genotype (*F*(1, 53) = 2.309, *P* = 0.1346). Post hoc comparisons using Šídák’s multiple comparisons test showed significant differences between several group pairs (WT V3W versus WT C3W, *P* < 0.0001; FGFR1-cKO V3W versus FGFR1-cKO C1W, *P* = 0.0115; FGFR1-cKO V3W versus FGFR1-cKO C3W, *P* = 0.0041). ns, not significant; *P < 0.05; **P < 0.01; ****P < 0.0001. **c** FGFR1 qRT–PCR results after behavior tests of **a**. The data are represented as means ± s.e.m.; *n* = 12 for V3W, *n* = 12 for C1W and *n* = 13 for C3W WT mice were included for each condition. A one-way ANOVA revealed a significant effects of corticosterone treatment (*F*(2, 34) = 28.31, *P* < 0.0001). Post hoc comparisons using Tukey’s multiple comparisons test showed significant differences between several group pairs (V3W versus C1W, *P* = 0.019; V3W versus C3W, *P* < 0.0001). *P < 0.05; ****P < 0.0001. **d** Western blot quantification for FGFR1 in WT mice after behavior tests. The data are represented as means ± s.e.m.; *n* = 18 for V3W, *n* = 14 for C1W and *n* = 17 for C3W WT mice were included for each condition. One-way ANOVA revealed a significant effect of corticosterone treatment (*F*(2, 46) = 47.44, *P* < 0.0001). Post hoc comparisons using Tukey’s multiple comparisons test showed significant differences between several group pairs (V3W versus C1W, *P* = 0.0003; V3W versus C3W, *P* < 0.0001.) ***P < 0.001; ****P < 0.0001. **e** A schematic representation and timeline and administration V3W and C1W and behavior tests in the region-specific FGFR1 knockout model. **f** Representative images showing Cre^+^ cells in different hippocampal regions in *Fgfr1*^*flox/flox*^ mouse indicating cKO of *Fgfr1* gene. Scale bar, 100 μm **g** Anxiety index, total immobility time in tail suspension test, and sucrose preference in WT mice and *Fgfr1*^*flox/flox*^ mice. The experimental groups included: WT; FGFR1-cKO mice, in which a Cre-virus was injected into the hippocampal CA1 of *Fgfr1*^*flox/flox*^ mice (FGFR1-cKO-CA1); FGFR1-cKO mice, in which a Cre-virus was injected into the hippocampal CA3 of *Fgfr1*^*flox/flox*^ mice (FGFR1-cKO-CA3); FGFR1-cKO mice, in which a Cre-virus was injected into the hippocampal dentate gyrus of *Fgfr1*^*flox/flox*^ mice (FGFR1-cKO-DG). Data are presented as means ± s.e.m.; *n* = 14 WT mice, *n* = 4 (FGFR1-cKO-CA1;V3W), *n* = 6 (FGFR1-cKO-CA1;C1W), *n* = 5 (FGFR1-cKO-CA3;V3W), *n* = 7 (FGFR1-cKO-CA3;C1W), *n* = 5 (FGFR1-cKO-DG;V3W), and *n* = 5 (FGFR1-cKO-DG;C1W) *Fgfr1*^*flox/flox*^ mice were included for each condition. For anxiety index, two-way ANOVA revealed a significant main effect of corticosterone treatment but no significant main effect of hippocampal subregion or interaction between treatment and subregion (interaction: *F*(2, 52) = 2.083, *P* = 0.1338; subregion: *F*(2, 52) = 0.3329, *P* = 0.7184; corticosterone treatment: *F*(1, 52) = 7.837, *P*= 0.0070). Post hoc comparisons using Tukey’s multiple comparisons showed no pairwise comparisons reached statistical significance. For immobility time, two-way ANOVA revealed a significant main effect of corticosterone treatment (*F*(3, 58) = 12.86, *P* < 0.0001) and a significant interaction between corticosterone treatment and hippocampal subregion (*F*(3, 58) = 9.256, *P* < 0.0001), but no significant main effect of hippocampal subregion alone (*F*(1, 58) = 3.979, *P* = 0.0508). Post hoc comparisons using Tukey’s multiple comparisons showed significant differences between a pair (FGFR1-cKO-DG V3W vs FGFR1-cKO-DG C1W, *P* < 0.0001). For sucrose preference, two-way ANOVA revealed a significant interaction between hippocampal subregion and corticosterone treatment (interaction: *F*(3, 53) = 15.31, *P* < 0.0001), as well as a significant main effect of corticosterone treatment (*F*(1, 53) = 13.44, *P* = 0.0006), but not of hippocampal subregion (*F*(3, 53) = 0.3939, *P* = 0.7579). Post hoc comparisons using Šídák’s multiple comparisons showed significant differences between several pairs (FGFR1-cKO-DG V3W vs FGFR1-cKO-DG C1W, *P* < 0.0001). ns, not significant; **** *p* < 0.0001. **h** Schematic representation of CUMS model of WT and *Fgfr1*^*flox*/*flox*^ mice. **i** FGFR1 qRT–PCR results after behavior tests of **h**. Data are represented as means ± s.e.m.; *n* = 6 WT mice were included for each condition. An unpaired two-tailed *t*-test revealed a significant increase in the CUMS group compared with controls (*t*(10) = 2.409, *P* = 0.0367). *P < 0.05. **j** Western blot quantification for FGFR1 in WT mice after behavior tests. Data are represented as means ± s.e.m.; *n* = 6 WT mice were included for each condition. An unpaired two-tailed *t*-test revealed a significant increase in the CUMS group compared with controls (*t*(10) = 3.246, *P* = 0.0088). **P < 0.01. **k** Body weight, anxiety index, total immobility time in tail suspension test, and sucrose preference of mouse subjected to CUMS. The experimental groups included: CUMS, a control group with no induced stress (control (Ctrl)) and FGFR1-cKO mice, in which a Cre-virus was injected into the hippocampal dentate gyrus of *Fgfr1*^*flox/flox*^ mice. The data are presented as means ± s.e.m.; *n* = 6 WT mice, *n* = 3 FGFR1-cKO Ctrl mice and *n* = 4 FGFR1-cKO-CUMS mice were included for each condition. For body weight, a two-way ANOVA revealed significant main effects of CUMS weeks (*F*(4, 75) = 11.33, *P* < 0.0001) and genotype (*F*(3, 75) = 52.80, *P* < 0.0001), as well as a significant interaction between CUMS weeks and genotype (*F*(12, 75) = 2.748, *P* = 0.0038). Post hoc comparisons using Tukey’s multiple comparisons test showed that FGFR1-cKO CUMS mice exhibited significantly reduced body weight compared with WT CTRL, WT CUMS and FGFR1-cKO Ctrl mice from week 1 onward (*P* < 0.01 to *P* < 0.0001). WT CUMS mice also showed significantly lower body weight than WT CTRL mice at week 3 (*P* = 0.0358) and week 4 (*P* = 0.0003). For anxiety index, a two-way ANOVA revealed no significant effects of CUMS weeks (*F*(4, 75) = 0.983, *P* = 0.4222), genotype (*F*(3, 75) = 1.992, *P* = 0.1225) or their interaction (*F*(12, 75) = 1.407, *P* = 0.1819). Post hoc comparisons using Tukey’s multiple comparisons test showed no pairwise comparisons reached statistical significance. For total immobility, two-way ANOVA revealed significant main effects of CUMS weeks (*F*(4, 75) = 22.80, *P* < 0.0001) and genotype (*F*(3, 75) = 37.59, *P* < 0.0001), as well as a significant interaction between CUMS weeks and genotype (*F*(12, 75) = 5.737, *P* < 0.0001). Post hoc comparisons using Tukey’s multiple comparisons test showed that FGFR1-cKO-CUMS mice displayed significant behavioral changes compared with FGFR1-cKO Ctrl mice, with significant differences from week 0 to weeks 1, 2, 3 and 4 (*P* < 0.05 to *P* < 0.0001). WT CUMS mice exhibited significant behavioral changes compared with WT CTRL, with significant differences observed at weeks 3 and 4 (*P* < 0.0001). WT CTRL and FGFR1-cKO Ctrl mice showed no significant differences across the time points (*P* > 0.05). For sucrose preference, a two-way ANOVA revealed significant main effects of CUMS weeks (*F*(4, 75) = 7.290, *P* < 0.0001) and genotype (*F*(3, 75) = 19.46, *P* < 0.0001), as well as a significant interaction between CUMS weeks and genotype (*F*(12, 75) = 3.394, *P* = 0.0005). Post hoc comparisons using Tukey’s multiple comparisons test showed that FGFR1-cKO-CUMS mice displayed significant behavioral changes compared with FGFR1-cKO Ctrl mice, with significant differences from week 0 to weeks 1, 2, 3 and 4 (*P* = 0.0146 to *P* = 0.0028). WT CUMS mice exhibited significant behavioral changes compared with WT CTRL, with significant differences observed at weeks 3 and 4 (*P* = 0.0002 to *P* < 0.0001). WT CTRL and FGFR1-cKO Ctrl mice showed no significant differences across the time points (*P* > 0.05). ns, not significant; *P < 0.05; **P < 0.01; ***P < 0.001; ****P < 0.0001.

We next evaluated the level of FGFR1 expression in mouse depressive models. First, to construct a chemically induced depressive model, we performed repeated corticosterone injections to wild-type (WT) mice, a method proven to elicit depressive-like behaviors^9^ (Fig. 2a). While the corticosterone administration did not alter the anxiety index or locomotor function of WT mice compared with the control group (Fig. 2b, left, and Supplementary Fig. 4a–c), changes in sucrose preferences and immobility time were observed compared with the WT vehicle group, confirming the presence of depressive-like behaviors (Fig. 2b, middle and right). No significant behavioral changes were observed in WT mice subjected to 1-week corticosterone administration, suggesting that chronic corticosterone exposure is necessary to induce depressive-like behaviors in WT mice. Interestingly, compared with the control group, both *Fgfr1* mRNA and FGFR1 protein levels significantly increased after 1 week of corticosterone administration and showed further elevation after 3 weeks (Fig. 2c, d). This pattern of FGFR1 upregulation prompted further investigation into its potential role before the full manifestation of the depressive phenotype.

To investigate the role of FGFR1 in the dentate gyrus in relation to depressive phenotype, we utilized the *Fgfr1*^*flox/flox*^ mice and an adeno-associated virus (AAV) expressing Cre-recombinase under Ca^2+^/calmodulin-dependent kinase IIα (CamKIIα) promotor, aiming to specifically eliminate the expression of FGFR1 in hippocampal dentate gyrus neurons (Fig. 2e, f and Supplementary Fig. 5a, b). Strikingly, these FGFR1 conditional knockout (cKO) mice exhibited depressive-like behaviors after just 1 week of corticosterone administration, without any discernible changes in anxiety index or locomotor function (Fig. 2b and Supplementary Fig. 4a–c). This suggests that the loss of FGFR1 in dentate gyrus neurons is associated with an accelerated onset of depressive-like behaviors, implying a potential role of FGFR1 as a protective factor against the onset of depression. Notably, the acceleration of depressive-like behaviors was unique to the dentate-gyrus-specific knockout of FGFR1 and was not observed in CA1- or CA3-specific knockouts (Fig. 2f, g). Similar to WT mice, corticosterone administration did not alter the anxiety index or locomotor function in region-specific FGFR1-cKO mice compared with the control group (Supplementary Fig. 4d–f).

We also adopted CUMS model^31^ to investigate whether the inverse correlation between FGFR1 expression and the onset of depressive-like behavior was similarly observed (Fig. 2h). Similar to the corticosterone-induced depression model, both *Fgfr1* mRNA and FGFR1 protein levels were significantly increased in the CUMS group mice 4 weeks after stress exposure (Fig. 2i, j). While CUMS did not affect the anxiety index or locomotor function in either WT or *Fgfr1*^*flox*/*flox*^ mice, alterations in body weight, sucrose preference and immobility time were observed (Fig. 2k and Supplementary Fig. 6a–g). As observed in the chemically inducible model, the depressive-like behavior parameters were significantly altered after just 1 week of CUMS in *Fgfr1*^*flox*/*flox*^ mice, compared with 3-week exposure required to elicit similar changes in WT mice. These results again suggest an accelerated onset of depressive-like behaviors in the absence of FGFR1 in the dentate gyrus.

Overall, our findings demonstrate that both corticosterone administration and CUMS-induced depressive-like phenotypes in mice are associated with elevated FGFR1 expression levels and that selective deletion of FGFR1 in the dentate gyrus accelerates the onset of depressive-like behaviors.

### A novel FGFR1–Notch–BDNF axis in dentate gyrus identified by optogenetics

To investigate FGFR1 signaling in hippocampal dentate gyrus neurons, we employed an optogenetically activatable FGFR1 (optoFGFR1), a tool that permits precise spatiotemporal activation of FGFR1 through its blue light-dependent oligomerization property^27^. We achieved the targeted expression of FGFR1 in granule neurons by introducing AAV-hSyn-DIO-optoFGFR1 into the dentate gyrus of CamKIIα-Cre mice (Supplementary Fig. 7a). Blue light stimulation (473 nm wavelength) of the optoFGFR1-expressing neurons led to a clear activation of downstream FGFR1 signaling pathways, specifically, phosphorylated Erk of the MAPK pathway and phosphorylated S6 of the Akt–mTOR pathway (Supplementary Fig. 7b–f). These results highlight that spatiotemporal activation of FGFR1 signaling through optogenetic techniques can reveal differential activation of signaling components over time.

**Fig. 3:**
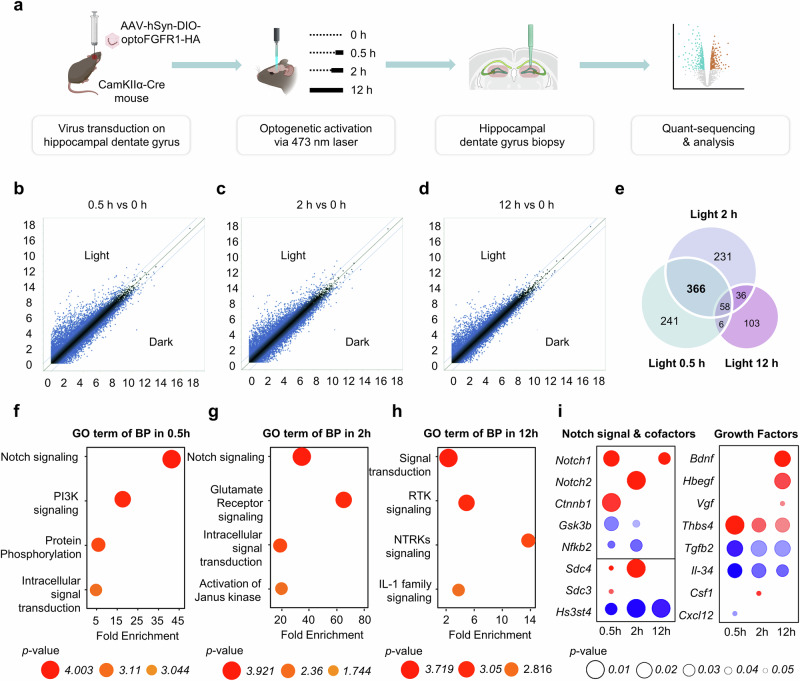
Spatiotemporal FGFR1 activation identifies a distinct activation of downstream components over time in the dentate gyrus. **a** A schematic representation of optoFGFR1 viral injection, activation tissue preparation, quant-sequencing and data analysis. **b**–**d** The scatter plots of 0.5 (**b**), 2 (**c**) and 12 h (**d**) FGFR1-activated genes versus 0 h control. **e** A Venn diagram of 0.5, 2 and 12 h of optoFGFR1 activation DEGs. **f**–**h** David analysis of fold enrichment in GO terms of biological process (BP), in 0.5 (**f**), 2 (**g**) and 12 h (**h**) FGFR1 activation. **i** A dot plot showing Notch signaling components, FGFR1 signaling cofactors and growth factors.

To obtain the spatiotemporal activation of FGFR1 in the dentate gyrus, we administrated AAV-hSyn-DIO-optoFGFR1 into the dentate gyrus of CamKIIα-Cre mice and exposed them to blue light illumination for 0, 0.5, 2 and 12 h (Fig. 3a). Transcriptomic analysis of the dentate gyrus revealed changes in gene expression, with 3234 genes differentially expressed at 0.5 h, 3291 genes at 2 h and 2,224 genes at 12 h post optoFGFR1 activation. These changes, including both upregulation and downregulation, are illustrated in the scatter plot (Fig. 3b–d). Notably, using the criteria of *P* value <0.05, fold change >2 and normalized data log_2_ >5, 257 DEGs were identified as upregulated and 414 downregulated at 0.5 h, 311 were upregulated and 380 downregulated at 2 h and 96 were upregulated and 107 downregulated at 12 h compared with the nonilluminated control. A total of 366 DEGs overlapped between the 0.5- and 2-h activations, while 58 DEGs were shared across the 0.5-, 2- and 12-h activations (Fig. 3e).

DAVID analysis showed that the Notch signaling pathway was highly ranked among the 671 genes activated at the 0.5-h FGFR1 activated group, along with PI3K and protein kinase B signaling (Fig. 3f). Similarly, the 691 genes from the 2-h FGFR1 activated group exhibited the Notch signaling pathway at the top rank, along with the glutamate receptor signaling pathway (Fig. 3g). Among the top-ranked pathways in the 12-h FGFR1 activated group, the NTRKs signaling pathway exhibited the highest fold enrichment (Fig. 3h). Given the significant enrichment of the Notch signaling pathway in both the 0.5-h and 2-h FGFR1 activated groups, we further explored Notch-related genes that were upregulated or downregulated. Notch1 was the most upregulated gene at 0.5 h, while among FGFR1 signaling cofactors, *Sdc4* was notably upregulated at 2 h (Fig. 3i). Also, growth factors genes such as *Bdnf*, *Hbegf* and *Vgf* were significantly upregulated in the 12-h FGFR1 activated group (Fig. 3i).

**Fig. 4:**
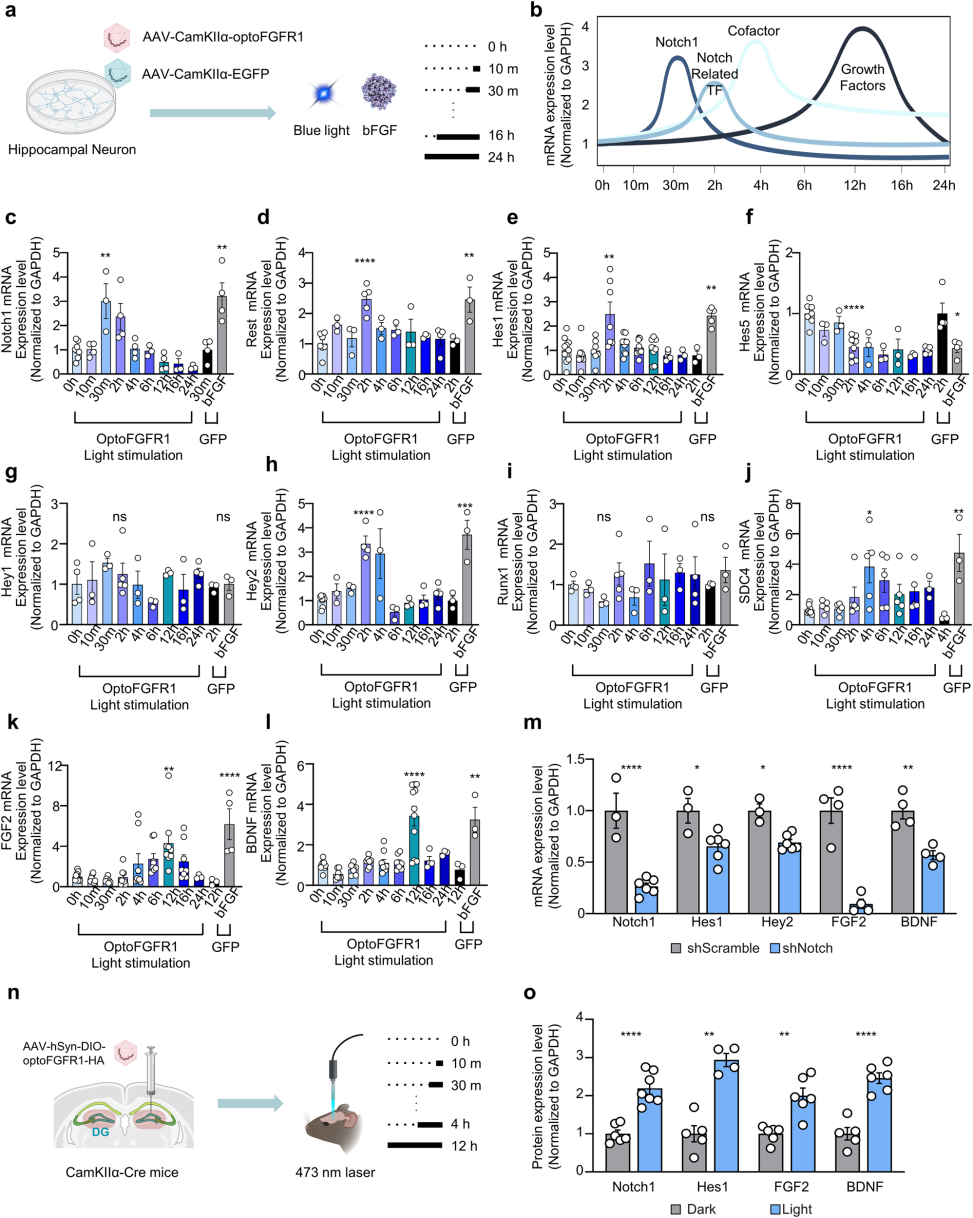
Dentate gyrus neuronal FGFR1 activation identifies a novel FGFR1–Notch–BDNF axis. **a** A schematic representation of viral transduction and activation in hippocampal neuronal culture. **b** A graphical summary of mRNA expression level upon FGFR1 activation. **c**–**l** qRT–PCR results for Notch1 (**c**), Rest (**d**), Hes1 (**e**), Hes5 (**f**), Hey1 (**g**), Hey2 (**h**), Runx1 (**i**), Sdc4 (**j**), FGF2 (**k**) and BDNF (**l**) in optoFGFR1-transduced neurons exposed to blue light illumination, GFP-transduced neurons were treated with soluble basic FGF. The data are presented as means ± s.e.m. The sample sizes were follow: for **c** *n* = 7 for 0 h light stimulation, *n* = 4 for 10 min light stimulation, *n* = 3 for 30 min light stimulation, *n* = 4 for 2 h light stimulation, *n* = 4 for 4 h light stimulation, *n* = 3 for 6 h light stimulation, *n* = 4 for 12 h light stimulation, *n* = 3 for 16 h light stimulation, *n* = 3 for 24 h light stimulation, *n* = 4 for 30 min light stimulation with EGFP expression and *n* = 4 for 30 min ligand treatment with EGFP expression. A one-way ANOVA revealed a significant effect of optoFGFR1 activation over time (*F*(10, 32) = 9.286, *P* < 0.0001). Post hoc comparisons using Tukey’s multiple comparisons test showed significant differences between control and optoFGFR1 activation (0 h versus 30 min, *P* = 0.003; 0 h versus bFGF, *P* = 0.0002; 30 min versus bFGF, *P* = 0.0012). For **d** *n* = 7 for 0 h light stimulation, *n* = 3 for 10 min light stimulation, *n* = 3 for 30 min light stimulation, *n* = 5 for 2 h light stimulation, *n* = 3 for 4 h light stimulation, *n* = 3 for 6 h light stimulation, *n* = 3 for 12 h light stimulation, *n* = 3 for 16 h light stimulation, *n* = 4 for 24 h light stimulation, *n* = 3 for 2 h light stimulation with EGFP expression and *n* = 3 for 2 h ligand treatment with EGFP expression. A one-way ANOVA revealed a significant effect of optoFGFR1 activation over time (*F*(10, 29) = 6.01, *P* < 0.0001). Post hoc comparisons using Tukey’s multiple comparisons test showed significant differences between control and optoFGFR1 activation (0 h versus 2 h, *P* < 0.0001; 0 h versus bFGF, *P* = 0.0011; 2 h versus bFGF, *P* = 0.0173). For **e** *n* = 11 for 0 h light stimulation, *n* = 7 for 10 min light stimulation, *n* = 7 for 30 min light stimulation, *n* = 7 for 2 h light stimulation, *n* = 9 for 4 h light stimulation, *n* = 9 for 6 h light stimulation, *n* = 8 for 12 h light stimulation, *n* = 6 for 16 h light stimulation, *n* = 3 for 24 h light stimulation, *n* = 3 for 2 h light stimulation with EGFP expression and *n* = 5 for 2 h ligand treatment with EGFP expression. A one-way ANOVA revealed a significant effect of optoFGFR1 activation over time (*F*(10, 64) = 7.018, *P* < 0.0001). Post hoc comparisons using Tukey’s multiple comparisons test showed significant differences between control and optoFGFR1 activation (0 h versus 2 h, *P* < 0.0001; 0 h versus bFGF, *P* = 0.001; 2 h versus bFGF, *P* = 0.0097). For **f** *n* = 7 for 0 h light stimulation, *n* = 3 for 10 min light stimulation, *n* = 3 for 30 min light stimulation, *n* = 8 for 2 h light stimulation, *n* = 3 for 4 h light stimulation, *n* = 3 for 6 h light stimulation, *n* = 3 for 12 h light stimulation, *n* = 3 for 16 h light stimulation, *n* = 5 for 24 h light stimulation, *n* = 4 for 2 h light stimulation with EGFP expression and *n* = 4 for 2 h ligand treatment with EGFP expression. A one-way ANOVA revealed a significant effect of optoFGFR1 activation over time (*F*(10, 35) = 8.159, *P* < 0.0001). Post hoc comparisons using Tukey’s multiple comparisons test showed significant differences between control and optoFGFR1 activation (0 h versus 2 h, *P* = 0.0002; 0 h versus bFGF, *P* = 0.0021; 2 h versus bFGF, *P* = 0.009). For **g** *n* = 4 for 0 h light stimulation, *n* = 3 for 10 min light stimulation, *n* = 3 for 30 min light stimulation, *n* = 5 for 2 h light stimulation, *n* = 3 for 4 h light stimulation, *n* = 3 for 6 h light stimulation, *n* = 3 for 12 h light stimulation, *n* = 3 for 16 h light stimulation, *n* = 4 for 24 h light stimulation, *n* = 3 for 2 h light stimulation with EGFP expression and *n* = 3 for 2 h ligand treatment with EGFP expression. A one-way ANOVA revealed no significant effect of optoFGFR1 activation over time (*F*(10, 26) = 1.016, *P* = 0.4571). Post hoc comparisons using Tukey’s multiple comparisons test showed no significant differences between control and optoFGFR1 activation pairs. For **h** *n* = 8 for 0 h light stimulation, *n* = 3 for 10 min light stimulation, *n* = 3 for 30 min light stimulation, *n* = 4 for 2 h light stimulation, *n* = 3 for 4 h light stimulation, *n* = 3 for 6 h light stimulation, *n* = 3 for 12 h light stimulation, *n* = 4 for 16 h light stimulation, *n* = 4 for 24 h light stimulation, *n* = 3 for 2 h light stimulation with EGFP expression and *n* = 3 for 2 h ligand treatment with EGFP expression. A one-way ANOVA revealed a significant effect of optoFGFR1 activation over time (*F*(10, 30) = 10.22, *P* < 0.0001). Post hoc comparisons using Tukey’s multiple comparisons test showed significant differences between control and optoFGFR1 activation (0 h versus 2 h, *P* < 0.0001; 0 h versus bFGF, *P* < 0.0001; 2 h versus bFGF, *P* = 0.0006). For **i** *n* = 4 for 0 h light stimulation, *n* = 3 for 10 min light stimulation, *n* = 3 for 30 min light stimulation, *n* = 5 for 2 h light stimulation, *n* = 3 for 4 h light stimulation, *n* = 3 for 6 h light stimulation, *n* = 3 for 12 h light stimulation, *n* = 3 for 16 h light stimulation, *n* = 4 for 24 h light stimulation, *n* = 3 for 2 h light stimulation with EGFP expression and *n* = 3 for 2 h ligand treatment with EGFP expression. A one-way ANOVA revealed no significant effect of optoFGFR1 activation over time (*F*(10, 26) = 0.7205, *P* = 0.6983). Post hoc comparisons using Tukey’s multiple comparisons test showed no significant differences between control and optoFGFR1 activation pairs. For **j** *n* = 8 for 0 h light stimulation, *n* = 5 for 10 min light stimulation, *n* = 5 for 30 min light stimulation, *n* = 5 for 2 h light stimulation, *n* = 5 for 4 h light stimulation, *n* = 5 for 6 h light stimulation, *n* = 5 for 12 h light stimulation, *n* = 5 for 16 h light stimulation, *n* = 3 for 24 h light stimulation, *n* = 3 for 4 h light stimulation with EGFP expression and *n* = 3 for 4 h ligand treatment with EGFP expression. A one-way ANOVA revealed a significant effect of optoFGFR1 activation over time (*F*(10, 41) = 3.781, *P* = 0.0012). Post hoc comparisons using Tukey’s multiple comparisons test showed significant differences between control and optoFGFR1 activation (0 h versus 4 h, *P* = 0.0212; 0 h versus bFGF, *P* = 0.0064; 4 h versus bFGF, *P* = 0.0139). For **k** *n* = 11 for 0 h light stimulation, *n* = 8 for 10 min light stimulation, *n* = 8 for 30 min light stimulation, *n* = 8 for 2 h light stimulation, *n* = 8 for 4 h light stimulation, *n* = 8 for 6 h light stimulation, *n* = 9 for 12 h light stimulation, *n* = 8 for 16 h light stimulation, *n* = 3 for 24 h light stimulation, *n* = 3 for 12 h light stimulation with EGFP expression and *n* = 4 for 12 h ligand treatment with EGFP expression. A one-way ANOVA revealed a significant effect of optoFGFR1 activation over time (*F*(10, 67) = 6.426, *P* < 0.0001). Post hoc comparisons using Tukey’s multiple comparisons test showed significant differences between control and optoFGFR1 activation (0 h versus 12 h, *P* = 0.002; 0 h versus bFGF, *P* < 0.0001; 12 h versus bFGF, *P* = 0.016). For **l** *n* = 11 for 0 h light stimulation, *n* = 7 for 10 min light stimulation, *n* = 7 for 30 min light stimulation, *n* = 7 for 2 h light stimulation, *n* = 9 for 4 h light stimulation, *n* = 7 for 6 h light stimulation, *n* = 11 for 12 h light stimulation, *n* = 4 for 16 h light stimulation, *n* = 3 for 24 h light stimulation, *n* = 3 for 12 h light stimulation with EGFP expression and *n* = 3 for 12 h ligand treatment with EGFP expression. A one-way ANOVA revealed a significant effect of optoFGFR1 activation over time (*F*(10, 61) = 12.08, *P* < 0.0001). Post hoc comparisons using Tukey’s multiple comparisons test showed significant differences between control and optoFGFR1 activation (0 h versus 12 h, *P* < 0.0001; 0 h versus bFGF, *P* = 0.001; 12 h versus bFGF, *P* = 0.0062). ns, not significant; *P < 0.05; **P < 0.01; ***P < 0.001; ****P < 0.0001. **m** qRT–PCR results for Notch1, Notch-related transcription factors and growth factors treated with shRNA-Scramble (gray) or shNotch1 (blue). Each component was optogenetically activated at its maximal expression time identified in **b**. The data are presented as means ± s.e.m.; *n* = 3 for Notch1 gene shScramble, *n* = 6 for Notch1 gene shRNA-Notch1, *n* = 3 for Hes1 gene shScramble, *n* = 6 for Hes1 gene shRNA-Notch1, *n* = 3 for Hey2 gene shScramble, *n* = 6 for Hey2 gene shRNA-Notch1, *n* = 4 for FGF2 gene shScramble, *n* = 4 for FGF2 gene shRNA-Notch1, *n* = 4 for BDNF gene shScramble and *n* = 4 for BDNF gene shRNA-Notch1. A two-way ANOVA revealed significant effect of genes (*F*(4, 33) = 6.035, *P* = 0.0009), shNotch1 (*F*(1, 33) = 130.8, *P* < 0.0001) and a significant interaction of the genes and shNotch1 (*F*(4, 33) = 6.035, *P* = 0.0009). Post hoc comparisons using Šídák’s multiple comparisons test showed that shRNA‐mediated Notch1 knockdown significantly reduced mRNA levels of Notch1 (*P* < 0.0001), Hes1 (*P* = 0.0137), Hey2 (*P* = 0.0322), FGF2 (*P* < 0.0001) and BDNF (*P* = 0.0015) compared with scramble control. *P < 0.05; **P < 0.01; ****P < 0.0001. **n** A schematic representation of optoFGFR1 viral injection and activation. **o** Western blots for Notch1, Hes1, FGF2 and BDNF using hippocampal dentate gyrus tissue lysate. Gray, no light activation; blue, light activation at each component’s maximal expression time identified in **b**. The data are presented as means ± s.e.m.; *n* = 6 for Notch1 protein dark, *n* = 7 for Notch1 protein light, *n* = 5 for Hes1 protein dark, *n* = 4 for Hes1 protein light, *n* = 6 for FGF2 protein dark, *n* = 6 for FGF2 protein light, *n* = 5 for BDNF protein dark and *n* = 6 for BDNF protein light. A two‐way ANOVA with target genes and light activation revealed significant main effects of light versus dark illumination (*F*(1, 37) = 153.1, *P* < 0.0001), target genes (*F*(3, 37) = 2.964, *P* = 0.0445) and a target genes and light activation interaction (*F*(3, 37) = 2.964, *P* = 0.0445). Post hoc comparisons using Šídák’s multiple comparisons test showed that light significantly increased levels of Notch1 (*P* < 0.0001), Hes1 (*P* < 0.0001), FGF2 (*P* = 0.0002) and BDNF (*P* < 0.0001) compared with dark controls. ***P < 0.001; ****P < 0.0001.

To further elucidate the temporal outcomes of FGFR1 activation, we employed primary hippocampal cultured neurons transduced with optoFGFR1 and activated them for various time periods (Fig. 4a). Through qRT–PCR, we validated the sequential elevation of Notch1, Notch-related transcription factors, FGFR1 signaling cofactors and growth factors (Fig. 4b). *Notch1* expression exhibited the elevation at 0.5-h FGFR1 activation (Fig. 4c), followed by Notch-related transcription factors such as *Rest*, *Hes1* and *Hey2* at 2-h FGFR1 activation (Fig. 4d–i). After 4-h FGFR1 activation, there was an induction in the expression of the cofactor *SDC4* (Fig. 4j), and ultimately, growth factors including *FGF2* and *BDNF* were elevated at 12-h FGFR1 activation (Fig. 4k, l). Knocking down the *Notch1* gene by treatment with *Notch1*-shRNA resulted in a concurrent decrease in the levels of Notch-related transcription factors, FGF2 and BDNF during their peak expression time (Fig. 4m), suggesting that preceding Notch activation by FGFR1 leads to the expression of the growth factors. The phenomenon was also identified in the mouse brain, where optoFGFR1 activation in the dentate gyrus resulted in an increased protein level of Notch1 at 0.5-h activation by blue light, Hes1 at 2-h activation and FGF2 and BDNF at 12-h activation (Fig. 4n, o). These findings underscore the existence of a novel FGFR1–Notch–BDNF axis in the hippocampal dentate gyrus and suggest a potential link between this axis and depressive-like behaviors.

### AHN induced by FGFR1–Notch–BDNF axis in dentate gyrus

**Fig. 5:**
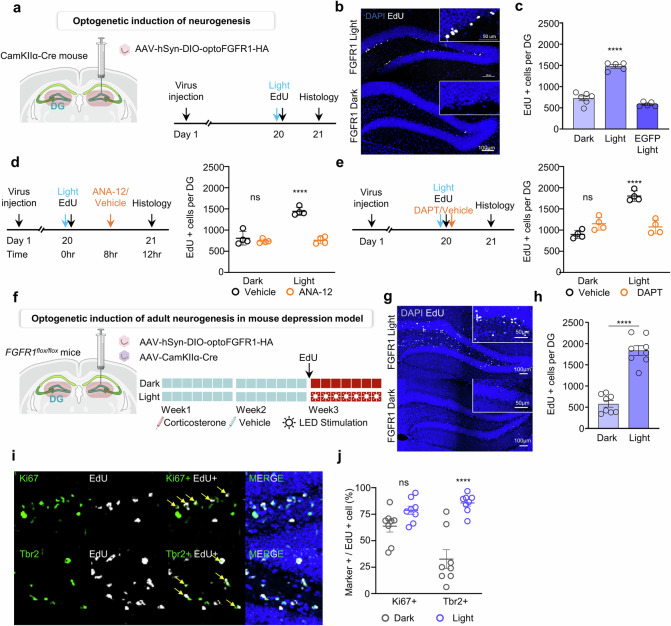
Optogenetic activation of FGFR1-induced AHN in depression model via FGFR1–Notch–BDNF axis in the dentate gyrus. **a** A schematic representation and timeline showing the viral injection in CamKIIα-Cre mice and administration of EdU with blue light. **b** Representation images showing EdU^+^ cell in subgranular zone (SGZ) of dentate gyrus under blue light stimulation for 12 h. Scale bar, 100 μm (inset, 50 μm). **c** A quantification of the data shown in **b**. The experimental groups included: CamKIIα-Cre mice injected with optoFGFR1 virus into the hippocampal dentate gyrus, kept in dark, and administered EdU 12 h before sacrifice (dark); CamKIIα-Cre mice injected with optoFGFR1 virus, given EdU and exposed to blue LED stimulation for 12 h (light); CamKIIα-Cre mice injected with EGFP control virus, given EdU and exposed to 12 h of blue LED stimulation (EGFP light). The data are represented as means ± s.e.m.; *n* = 6 (dark), *n* = 5 (light) and *n* = 5 (EGFP light), CamKIIα-Cre mice were included for each condition. A one-way ANOVA revealed a significant effect of optoFGFR1 light stimulation (*F*(2,13) = 107.8, *P* < 0.0001). Post hoc comparisons using Tukey’s multiple comparisons test showed that light had significantly more EdU^+^ cells than both the dark (*P* < 0.0001) and EGFP light (*P* < 0.0001). ****P < 0.0001. **d** An experimental timeline for evaluating neural stem cell proliferation following blockade of the BDNF-TrkB pathway in optoFGFR1-transduced mouse hippocampus (left) and quantification of EdU^+^ cells in the SGZ after 12 h of blue light stimulation (right). The data are represented as means ± s.e.m.; *n* = 4 mice were included for each condition. A two-way ANOVA revealed significant main effects of ANA-12 treatment (*F*(1,12) = 52.00, *P* < 0.0001), light stimulation (*F*(1,12) = 39.20, *P* < 0.0001) and their interaction (*F*(1,12) = 35.00, *P* < 0.0001). Post hoc comparisons using Šídák’s multiple comparisons test showed that light stimulation significantly increased EdU^+^ cell numbers in vehicle-treated mice compared with dark controls (*P* < 0.0001), while ANA-12 treatment blocked this effect (light vehicle versus light ANA-12, *P* < 0.0001). No significant differences were found within dark conditions or between ANA-12–treated groups under light exposures. ns, not significant; ****P < 0.0001. **e** An experimental timeline for evaluating neural stem cell proliferation following blockade of the Notch pathway via gamma-secretase inhibition in optoFGFR1-transduced mouse hippocampus (left) and quantification of EdU^+^ cells in the SGZ after 12 h of blue light stimulation (right). The data are represented as means ± s.e.m.; *n* = 4 mice were included for each condition. A two-way ANOVA revealed significant main effects of DAPT treatment (*F*(1,12) = 12.37, *P* = 0.0043), light stimulation (*F*(1,12) = 37.26, *P* < 0.0001), and their interaction (*F*(1,12) = 52.30, *P* < 0.0001). Post hoc comparisons using Šídák’s multiple comparisons test showed that light stimulation significantly increased EdU^+^ cell numbers in vehicle-treated mice compared with dark controls (*P* < 0.0001), while DAPT treatment blocked this effect (Light Vehicle vs Light DAPT, *P* < 0.0001). No significant differences were found within dark conditions or between DAPT–treated groups under light exposures. ns, not significant; ****P < 0.0001. **f** Schematic representation and timeline showing optogenetic induction of adult neurogenesis in mouse depression model. The viral injection of *Fgfr1*^*flox*/*flox*^ mice undergo administration of vehicle for 2 weeks followed by EdU and 1 week of corticosterone with blue light. **g** Representative images showing EdU^+^ cell in SGZ after administration of vehicle and corticosterone for 3 weeks. Scale bar, 100 μm (inset, 50 μm). **h** Quantification of the data shown in **g**. Data are represented as means ± s.e.m.; *n* = 8 mice were included for each condition. An unpaired two-tailed *t*-test revealed a significant increase in proliferating cells in the light group(*t*(14) = 9.182, *P* < 0.0001). **** *P* < 0.0001. **i** Representative images of EdU^+^ cells counterstained for Ki-67 (upper) and Tbr2 (lower). Arrows indicate cells with colocalizing signals. Scale bars, 50 μm. **j** Quantification of the data shown in **i**. Data are represented as means ± s.e.m.; *n* = 8 mice were included for each condition. Two-way ANOVA revealed significant main effects of light stimulation (*F*(1,28) = 34.39, *P* < 0.0001), marker type (*F*(1,28) = 4.337, *P* = 0.0465), and their interaction (*F*(1,28) = 10.80, *P* = 0.0027). Post hoc comparisons using Šídák’s multiple comparisons test showed that light stimulation significantly increased the number of Tbr2+ cells compared with dark controls (*P* < 0.0001), while no significant difference was observed in KI67^+^ cells between light and dark groups (*P* = 0.1517). ns, not significant; ****P < 0.0001.

We then investigated the downstream effects of BDNF upregulation through activation of the FGFR1–Notch–BDNF axis, which can contribute to the depressive phenotype. One of the most extensively studied mechanisms by which BDNF exerts its antidepressant effects is through the enhancement of AHN^32,33^. To assess neural stem cell proliferation in the dentate gyrus, we administered AAV-hSyn-DIO-optoFGFR1 into the dentate gyrus of CamKIIα-Cre mice and exposed them to blue light illumination for 12-h in the presence of EdU (Fig. 5a). IHC analysis of EdU incorporation revealed that 12-h optoFGFR1 activation significantly increased the number of EdU^+^ cells compared with the control group (Fig. 5b, c), suggesting enhanced neural stem cell proliferation in the dentate gyrus.

To determine whether the FGFR1–Notch–BDNF axis is responsible for this neurogenic effect, we pharmacologically inhibited BDNF-TrkB signaling by administering ANA-12, a selective TrkB inhibitor, in optoFGFR1-activated mice (Fig. 5d). ANA-12 treatment effectively blocked neural stem cell proliferation induced by optoFGFR1 activation via light stimulation. Furthermore, inhibition of EdU^+^ cell proliferation in the FGFR1-activated group was also observed following treatment with DAPT, a Notch signaling pathway inhibitor (Fig. 5e). These findings underscore that the observed increase in neurogenesis is mediated by activation of the FGFR1–Notch–BDNF axis in the dentate gyrus.

We also examined the optoFGFR1-induced AHN in the mouse depression model. To achieve this, we administered AAV-hSyn-DIO-optoFGFR1 and AAV-CamKIIα-Cre into the dentate gyrus of *Fgfr1*^*flox/flox*^ mice, followed by either vehicle or corticosterone administration with blue light illumination for 1 week in the presence of EdU (Fig. 5f). IHC analysis revealed that optoFGFR1-activated mice exhibited a significant increase in EdU^+^ cells compared with mice maintained in dark condition (Fig. 5g, h). Further characterization through IHC analysis of Ki67 and Tbr2 suggested that the increased EdU^+^ cells in the FGFR1-activated group were predominantly intermediate progenitor cells^34^ (Fig. 5i, j).

### Antidepressive role of FGFR1–Notch–BDNF axis in dentate gyrus

Importantly, the increase in neural stem cell population correlated with a rescue of depressive-like behaviors in FGFR1-cKO mice. While the anxiety index remained unchanged (Supplementary Fig. 8a), optogenetic activation of FGFR1 in the dentate gyrus for 12 h per day over 1 week—sufficient to fully activate the FGFR1–Notch–BDNF axis—resulted in a significant increase in sucrose preference and reduction in total immobility time (Supplementary Fig. 8b, c). These behavioral improvements imply that FGFR1-mediated neurogenesis specifically alleviates depressive-like phenotypes.

**Fig. 6:**
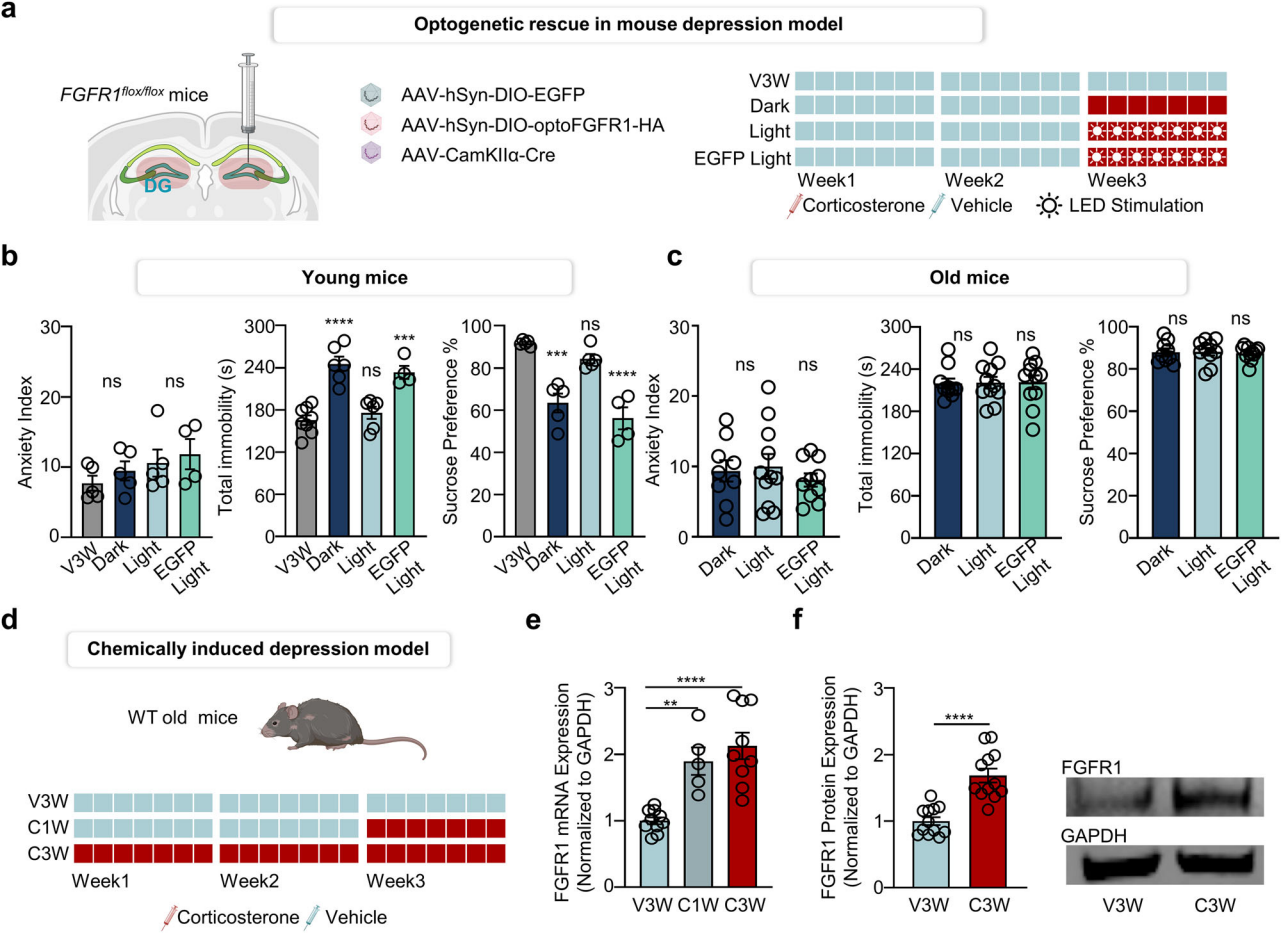
Optogenetic rescue of young mouse depression model via the FGFR1–Notch–BDNF axis in the dentate gyrus. **a** A schematic representation and timeline showing the viral injection in *Fgfr1*^*flox/flox*^ mice and administration of corticosterone with blue light. **b** Anxiety index, total immobility and sucrose preference in *Fgfr1*^*flox/flox*^ mice. The experimental groups included: FGFR1-cKO mice with V3W, in which a Cre virus was injected into the hippocampal dentate gyrus of *Fgfr1*^*flox/flox*^ mice (V3W); FGFR1-cKO mice, in which an optoFGFR1 virus was injected into the hippocampal dentate gyrus of *Fgfr1*^*flox/flox*^ mice with C1W under dark room condition (dark); FGFR1-cKO mice, in which an optoFGFR1 virus was injected into the hippocampal dentate gyrus of *Fgfr1*^*flox/flox*^ mice with C1W and blue LED stimulation for 7 days (light); FGFR1–EGFP mice, in which a EGFP-virus (control virus) was injected into the hippocampal dentate gyrus of *Fgfr1*^*flox/flox*^ mice followed by C1W and blue LED stimulation for 7 days (EGFP light). The data are presented as means ± s.e.m.; *n* = 5 (FGFR1-cKO;V3W), *n* = 5 (FGFR1-cKO; dark), *n* = 5 (FGFR1-cKO; light), *n* = 4 (FGFR1–EGFP; EGFP light) *Fgfr1*^*flox/flox*^ mice were included for each condition. For anxiety index, a one-way ANOVA revealed no significant effect on anxiety index (*F*(3,15) = 1.11, *P* = 0.3758). Post hoc comparisons using Tukey’s multiple comparisons test showed no significant differences between any groups (all *P* > 0.05). For total immobility, one-way ANOVA revealed a significant main effect on immobility time in the tail suspension test (*F*(3,20) = 21.77, *P* < 0.0001). Post hoc comparisons using Tukey’s multiple comparisons test showed that immobility time was significantly increased in dark compared with V3W (*P* < 0.0001) and light significantly reduced immobility compared with dark (*P* < 0.0001). EGFP light also significantly increased immobility compared with light (*P* = 0.0025), while no significant difference was found between dark and EGFP light (*P* = 0.8268) or between V3W and light (*P* = 0.7943). For sucrose preference, a one-way ANOVA revealed a significant main effect of sucrose preference (*F*(3,15) = 23.43, *P* < 0.0001). Post hoc comparisons using Tukey’s multiple comparisons test showed that the V3W exhibited significantly higher preference compared with the dark (*P* < 0.0001) and EGFP light (*P* < 0.0001). Dark showed significantly lower preference compared with light (*P* = 0.0025), while no significant difference was observed between the dark and EGFP light (*P* = 0.4867) or between the V3W and light (*P* = 0.4264). ns, not significant; ***P < 0.001; ****P < 0.0001. **c** Anxiety index, total immobility and sucrose preference in old *Fgfr1*^*flox/flox*^ mice. The experimental groups included: old FGFR1-cKO mice, in which an optoFGFR1 virus was injected into the hippocampal dentate gyrus of *Fgfr1*^*flox/flox*^ mice with C1W under dark room condition (dark); old FGFR1-cKO mice, in which an optoFGFR1 virus was injected into the hippocampal dentate gyrus of *Fgfr1*^*flox/flox*^ mice with C1W and blue LED stimulation for 7 days (light); old FGFR1–EGFP mice, in which a EGFP-virus (control virus) was injected into the hippocampal dentate gyrus of *Fgfr1*^*flox/flox*^ mice followed by C1W and blue LED stimulation for 7 days (EGFP light). The data are presented as means ± s.e.m.; *n* = 9 (dark); *n* = 11 (light); and *n* = 10 (EGFP Light) old *Fgfr1*^*flox/flox*^ mice were included for each condition. For the Old-FGFR1 anxiety index, a one-way ANOVA revealed no significant main on anxiety index (*F*(2,27) = 0.4555, *P* = 0.6389). Post hoc comparisons using Tukey’s multiple comparisons test showed no significant differences between the dark and light (*P* = 0.9526), Dark and EGFP light (*P* = 0.8187) or light and EGFP light (*P* = 0.6193). For the Old-FGFR1 total immobility, one-way ANOVA revealed no significant main effect on immobility time (*F*(2,29) = 0.01364, *P* = 0.9865). Post hoc comparisons using Tukey’s multiple comparisons test showed no significant differences between the dark and light (*P* = 0.9926), dark and EGFP light (*P* = 0.9858) or light and EGFP light (*P* = 0.9988). For the Old-FGFR1 sucrose preference, one-way ANOVA revealed no significant main on preference (*F*(2,27) = 0.0674, *P* = 0.935). Post hoc comparisons using Tukey’s multiple comparisons test showed no significant differences between the dark and light (*P* = 0.9966), dark and EGFP light (*P* = 0.9634) or light and EGFP light (*P* = 0.9349). ns, not significant. **d** A schematic representation and timeline showing the administration of vehicle and corticosterone in WT old mice. **e** FGFR1 qRT–PCR results in vehicle- or corticosterone- administrated mice after behavior tests. The data are represented as means ± s.e.m.; *n* = 10 for old V3W, *n* = 5 for old C1W and *n* = 9 for old C3W mice. A one-way ANOVA revealed a significant main effect of corticosterone treatment (*F*(2,21) = 17.44, *P* < 0.0001). Post hoc comparisons using Tukey’s multiple comparisons test showed that the both the C1W group (*P* = 0.003) and the C3W group (*P* < 0.0001) had significantly higher FGFR1 mRNA levels compared with V3W. No significant difference was observed between the C1W and C3W groups (*P* = 0.6152). **P < 0.01; ****P < 0.001. **f** Western blot image and quantification for FGFR1 and GAPDH in vehicle- or corticosterone-administrated mice after behavior tests. The data are represented as means ± s.e.m.; *n* = 12 old WT mice were included for each condition. An unpaired two-tailed *t*-test revealed a significant difference between the V3W and C3W groups (*t*(22) = 5.718, *P* < 0.0001). ****P < 0.0001.

Interestingly, we observed that the antidepressant effects of FGFR1–Notch–BDNF–AHN axis activation were effective only in young mice (12 week old) (Fig. 6a, b and Supplementary Fig. 4g–i) but not in elderly mice (over 1 year old). Aged FGFR1-cKO mice did not exhibit a rescue of depressive-like behavior following optogenetic activation of FGFR1 signaling (Fig. 6c and Supplementary Fig. 4j–l). However, the increase in FGFR1 expression during the first week of corticosterone administration was similarly observed in elderly WT mice (Fig. 6d–f), suggesting the presence of a potential inhibitory mechanism that limits the effectiveness of FGFR1–Notch–BDNF axis activation in aged individuals.

### Age-related dysregulation of FGFR1 signaling in MDD

**Fig. 7:**
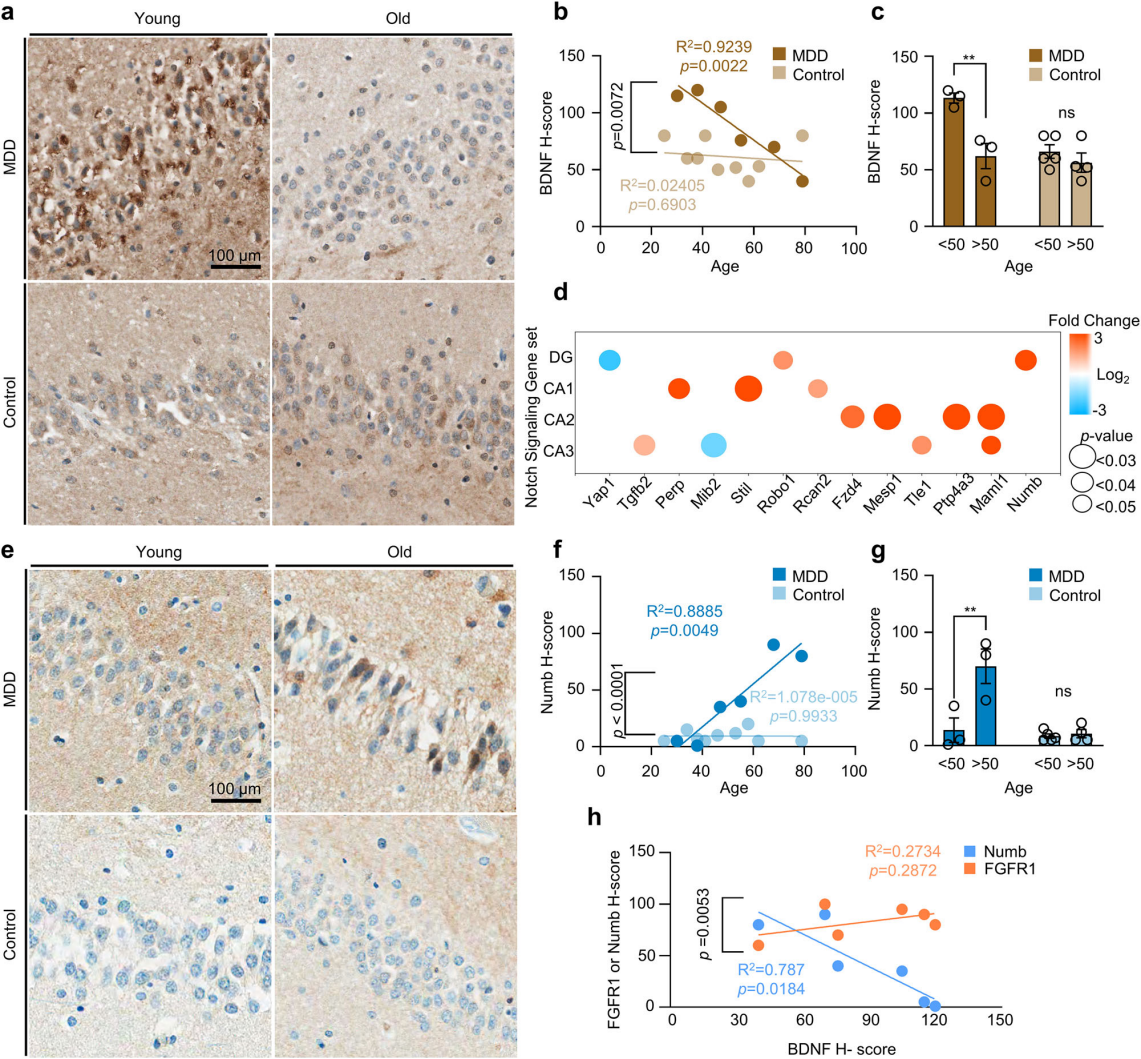
Age-related dysregulation of FGFR1 signaling contributes to biochemical hallmarks of progressive depression. **a** Representative images showing the expression of BDNF in human patients with MDD and normal control dentate gyrus. Scale bar, 100 μm. **b** Distribution of BDNF *H*-score according to age in patients with MDD and normal control individuals. Data are represented as best-fit values ± standard error; samples included *n* = 6 for normal control and *n* = 3 for with MDD in each group. A linear regression analysis revealed a significant negative correlation between the *x* and *y* values in the MDD group (slope −1.62, 95% confidence interval (CI) −2.27 to −0.97; *F*(1,4) = 48.55, *P* = 0.0022; *R*² = 0.9239), indicating a strong linear relationship. By contrast, no significant correlation was observed in the control group (slope −0.1412, 95% CI −0.9448 to 0.6625; *F*(1,7) = 0.1725, *P* = 0.6903; *R*² = 0.02405). ns, not significant; **P < 0.01. **c** A quantification of BDNF *H*-score according to age groups showing decreased expression of BDNF in patients with MDD as their age is over 50 years. The data are represented as means ± s.e.m.; samples included *n* = 5 for normal control under 50 years old, *n* = 4 for normal control over 50 years old, *n* = 3 for MDD under 50 years old and *n* = 3 for MDD over 50 years old. A two-way ANOVA revealed significant main effects of both group (MDD versus control; (*F*(1,11) = 15.08, *P* = 0.0025) and age (<50 versus >50 years; *F*(1,11) = 11.39, *P* = 0.0062), as well as a significant interaction between factors (*F*(1,11) = 6.988, *P* = 0.0228). Post hoc comparisons using Šídák’s multiple comparisons test showed that in the MDD group, <50 participants had significantly higher BDNF *H*-scores than >50 participants (mean difference 51.33, 95% CI 19.90 to 82.76, *P* = 0.0029), while no significant difference was found between age groups in the control group (*P* = 0.5773). ns, not significant; **P < 0.01. **d** A dot plot showing expression levels of the Notch signaling genes in each region with filtered gene sets of the hippocampal subregions of patients with MDD compared with normal control. **e** Representative images showing the expression of Numb in human patients’ with MDD and healthy individuals’ dentate gyri. Scale bar, 100 μm. **f** Distribution of Numb *H*-score according to age in patients with MDD and healthy individuals. The data are represented as best-fit values ± standard error; samples included *n* = 6 for normal control and *n* = 3 for with MDD in each group. Linear regression analysis revealed a significant positive correlation between age and Numb *H*-score values in the MDD (slope 1.895, 95% CI 0.9629 to 2.827; *F*(1,4) = 31.86, *P* = 0.0049; *R*² =0.8885), indicating a strong linear relationship. By contrast, no significant correlation was observed in the control (slope −0.001081, 95% CI −0.2954 to 0.2932; *F*(1,7) = 0.00007546, *P* = 0.9933; *R*² = 0.00001). ns, not significant; **P < 0.01. **g** A quantification of Numb *H*-score according to age groups showing decreased expression of Numb in patients with MDD as their age is over 50 years. The data are represented as means ± s.e.m.; samples included *n* = 5 for normal control under 50 years old, *n* = 4 for normal control over 50 years old, *n* = 3 for MDD under 50 years old and *n* = 3 for MDD over 50 years old. Two-way ANOVA revealed significant main effects of both group (MDD versus control; *F*(1,11) = 17.85, *P* = 0.0014) and age (<50 versus >50; *F*(1,11) = 14.53, *P* = 0.0029), as well as a significant interaction between factors (*F*(1,11) = 12.52, *P* = 0.0047). Post hoc comparisons using Šídák’s multiple comparisons test showed that Numb *H*-score was significantly higher in >50 versus <50 years within the MDD (mean difference −56.33, 95% CI −86.96 to −25.70, *P* = 0.0012) but not in the control (*P* = 0.9721). ns, not significant; **P < 0.01. **h** Distribution of FGFR1 or Numb *H*-score according to BDNF *H*-score showing negative correlation of Numb *H*-score as BDNF *H*-score increases. The data are represented as best-fit values ± standard error; *n* = 6 for MDD human samples were included for each group. A linear regression analysis revealed a significant negative correlation between Numb (blue) *H*-score and BDNF *H*-score (slope −1.058, 95% CI −1.823 to −0.2941; *F*(1,4) = 14.78, *P* = 0.0184; *R*² = 0.787), indicating a strong linear relationship. By contrast, no significant correlation was observed for FGFR1 (orange) (slope 0.2597, 95% CI −0.3281 to 0.8475; *F*(1,4) = 1.505, *P* = 0.2872; *R*² = 0.2734). ns, not significant; *P < 0.05.

Consistent with findings from the mouse model, the IHC analysis of human tissue revealed that BDNF expression was notably higher in young patients with MDD, decreasing with age (Fig. 7a, b). By contrast, BDNF levels in normal controls remained relatively constant over age, though at lower levels compared with young patients with MDD. A significant difference in BDNF expression was observed between age groups of patients with MDD, with a stratification threshold at age 50 years (Fig. 7c). This trend was also reflected in the mouse depression model, where BDNF mRNA levels were elevated in young WT mice but showed no significant differences in older mice (Supplementary Fig. 9a, b).

**Fig. 8:**
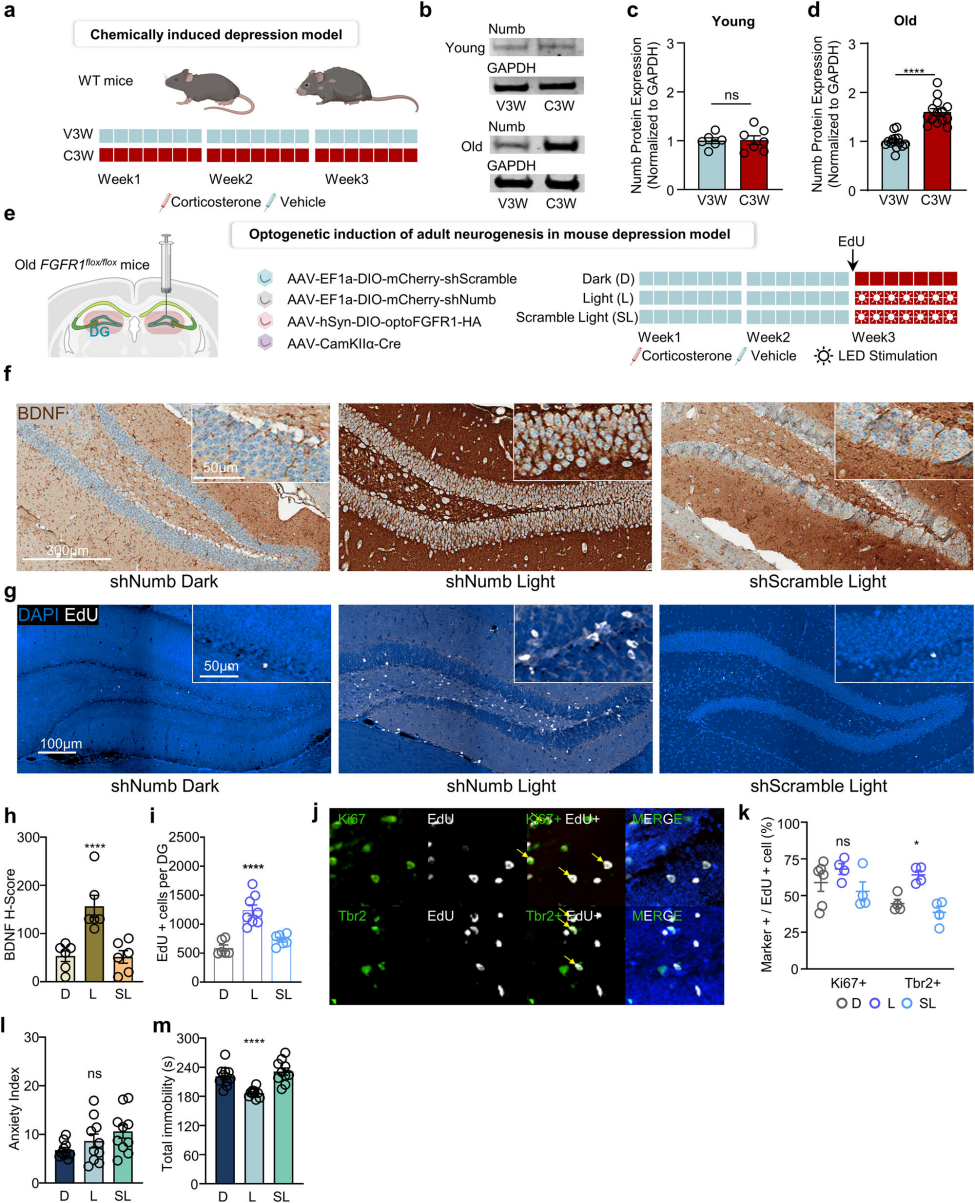
Targeting age-related FGFR1 dysregulation promotes AHN and reverses depressive phenotype in aged mice. **a** A schematic representation and timeline showing the administration of vehicle and corticosterone in WT young and old mice. V3W, administration of vehicle for 3 weeks; C3W, administration of corticosterone for 3 weeks. **b** Western blot for FGFR1 and GAPDH in vehicle- or corticosterone-administrated young and old mice. **c** A quantification of FGFR1 to GAPDH from the young groups in **b** showing no difference between the C3W and the V3W groups. The data are represented as means ± s.e.m.; *n* = 6 for V3W and *n* = 7 for C3W mice. An unpaired two-tailed *t*-test showed no significant difference in Numb expression between the C3W and V3W in young WT mice (*t*(11) = 0.1177, *P* = 0.9085). ns, not significant. **d** A quantification of FGFR1 to GAPDH from the old group in **b** showing a significant increase in the C3W group compared with the V3W group. The data are represented as means ± s.e.m.; *n* = 14 mice were included for each condition. An unpaired two-tailed *t*-test revealed a significant increase in Numb expression in the C3W compared with the V3W in old WT mice (*t*(26) = 7.574, *P* < 0.0001).****P < 0.0001; ns, not significant. **e** A schematic representation and timeline showing optogenetic induction of adult neurogenesis in mouse depression model. The viral injection in old *Fgfr1*^*flox/flox*^ mice and administration of vehicle for 2 weeks, followed by EdU and 1 week of corticosterone with blue light then behavior tests. D, L and SL represent the AAV-EF1a-DIO-mCherry-shNumb injection kept in dark, AAV-EF1a-DIO-mCherry-shNumb with light stimulation and AAV-EF1a-DIO-mCherry-shScramble injection with light stimulation, respectively. **f** Representative immunohistochemical staining images showing BDNF expression in the dentate gyrus of mice injected with AAV-EF1a-DIO-mCherry-shNumb and kept in the dark, AAV-EF1a-DIO-mCherry-shNumb with light stimulation and AAV-EF1a-DIO-mCherry-shScramble with light stimulation. Scale bar, 300 μm; 50 μm (inset). **g** Representative images showing EdU^+^ expression in the dentate gyrus of mice injected with AAV-EF1a-DIO-mCherry-shNumb and kept in the dark, AAV-EF1a-DIO-mCherry-shNumb with light stimulation and AAV-EF1a-DIO-mCherry-shScramble with light stimulation. Scale bar, 100 μm; 50 μm (inset). **h** A quantification of BDNF *H*-score according to BDNF expression in the dentate gyrus of mice injected with AAV-EF1a-DIO-mCherry-shNumb and kept in the dark, AAV-EF1a-DIO-mCherry-shNumb with light stimulation and AAV-EF1a-DIO-mCherry-shScramble with light stimulation. D, L, and SL represent the AAV-EF1a-DIO-mCherry-shNumb injection kept in dark, AAV-EF1a-DIO-mCherry-shNumb with light stimulation and AAV-EF1a-DIO-mCherry-shScramble injection with light stimulation, respectively. The data are represented as means ± s.e.m.; *n* = 6 mouse samples were included for each condition. A one-way ANOVA revealed a significant effect on BDNF expression (*F*(2, 15) = 13.37, *P* = 0.0005). Post hoc comparisons using Tukey’s multiple comparisons test showed significant differences between D and L (*P* = 0.0013) and between L and SL (*P* = 0.0011) but not between D and SL (*P* = 0.9972). **P < 0.01. **i** A quantification of EdU^+^ cells in the subgranular zone of dentate gyrus in **g**. The data are represented as means ± s.e.m.; *n* = 6 for D, *n* = 8 for L and *n* = 6 for SL. A one-way ANOVA revealed a significant effect on EdU⁺ cell counts (*F*(2, 17) = 22.98, *P* < 0.0001). Post hoc comparisons using Tukey’s multiple comparisons test showed significant differences between D and L (*P* < 0.0001) and between L and SL (*P* = 0.0004) but not between D and SL (*P* = 0.4133). ****P < 0.0001. D, L and SL represent the AAV-EF1a-DIO-mCherry-shNumb injection kept in dark, AAV-EF1a-DIO-mCherry-shNumb with light stimulation and AAV-EF1a-DIO-mCherry-shScramble injection with light stimulation, respectively. **j** Representative images of EdU^+^ cells counterstained for Ki-67 (top) and Tbr2 (bottom). The arrows indicate cells with colocalizing signals. Scale bars, 50 μm. **k** A quantification of the data shown in **j**. The data are represented as means ± s.e.m. The sample sizes were as follow: *n* = 6 for Ki-67 D, *n* = 4 for Ki-67 L, *n* = 4 for Ki-67 SL, *n* = 4 for Tbr2 D, *n* = 4 for Tbr2 L and *n* = 4 for Tbr2 SL. A two-way ANOVA revealed significant main effects of light stimulation (*F*(2,20) = 8.244, *P* = 0.0024) and marker type (*F*(1,20) = 6.979, *P* = 0.0156) but no significant interaction between light stimulation and marker type (*F*(2,20) = 0.6860, *P* = 0.5151). Post hoc comparisons using Šídák’s multiple comparisons test showed a significant increase in Tbr2^+^ cells under the L compared with SL (*P* = 0.0138), while no significant difference was observed between D and L (*P* = 0.7004) or between D and SL (*P* = 0.9446) in KI67^+^ cells. ns, not significant; *P < 0.05. D, L and SL represent the AAV-EF1a-DIO-mCherry-shNumb injection kept in dark, AAV-EF1a-DIO-mCherry-shNumb with light stimulation and AAV-EF1a-DIO-mCherry-shScramble injection with light stimulation, respectively. **l** Anxiety index in optogenetically rescued aged *Fgfr1*^*flox/flox*^ mice with cKO in DG. D, L and SL represent the AAV-EF1a-DIO-mCherry-shNumb injection kept in dark, AAV-EF1a-DIO-mCherry-shNumb with light stimulation and AAV-EF1a-DIO-mCherry-shScramble injection with light stimulation, respectively. The data are presented as means ± s.e.m.; *n* = 10 mice were included for each condition. A one-way ANOVA revealed no significant effect on anxiety index (*F*(2,27) = 2.579, *P* = 0.0944). Post hoc comparisons using Tukey’s multiple comparisons test showed no significant differences between any groups (all *P* > 0.05). ns, not significant. **m** Total immobility in optogenetically rescued aged *Fgfr1*^*flox/flox*^ mice with cKO in dentate gyrus. D, L and SL represent the AAV-EF1a-DIO-mCherry-shNumb injection kept in dark, AAV-EF1a-DIO-mCherry-shNumb with light stimulation and AAV-EF1a-DIO-mCherry-shScramble injection with light stimulation, respectively. The data are presented as means ± s.e.m.; *n* = 10 mice were included for each condition. A one-way ANOVA revealed a significant main effect on immobility time in the tail suspension test (*F*(2,27) = 15.63, *P* < 0.0001). Post hoc comparisons using Tukey’s multiple comparisons test showed that immobility time was significantly reduced in L compared with D (*P* = 0.0008) and L compared with SL (*P* < 0.0001), while no significant difference was found between D and SL (*P* = 0.4857). ****P < 0.0001.

We therefore speculated the presence of an age-dependent expression of a potential element that might inhibit the FGFR1-Notch1-BDNF signaling axis. Analysis of transcriptomic data from older patients with MDD (aged 55, 68 and 79 years) showed a marked increase in the expression of Numb, a protein known to suppress Notch signaling^35^, particularly in the dentate gyrus (Fig. 7d). IHC further demonstrated that Numb expression was higher in the dentate gyrus of older patients with MDD compared with younger patients or healthy controls, with Numb levels increasing with age in patients with MDD. (Fig. 7e–g). BDNF levels and Numb levels showed an inverse correlation in patients with MDD, whereas no significant correlation was observed between FGFR1 and BDNF (Fig. 7h). The elevated Numb expression was also identified in the corticosterone-induced old depressive mice but not in young mice (Fig. 8a–d).

To determine whether downregulating Numb could reinstate the FGFR1-Notch1-BDNF axis and alleviate depressive-like behaviors in aged mice, we administered AAV-CamKIIα-Cre and AAV-hSyn-DIO-optoFGFR1 to the *Fgfr1*^*flox/flox*^ old mice, along with either AAV-EF1α-DIO-mCherry-shNumb or AAV-EF1α-DIO-mCherry-shScramble (Supplementary Fig. 10a, b). Mice were then subjected to 1 week of corticosterone administration to induce depression, followed by optogenetic activation of FGFR1 in the dentate gyrus and EdU labeling (Fig. 8e). Histological analysis of the dentate gyrus revealed that BDNF expression was increased only in FGFR1-activated mice with Numb knockdown (shNumb light; L) but not in FGFR1-inactive mice with Numb knockdown (shNumb dark; D) or in FGFR1-activated mice without Numb knockdown (shScramble light; SL) (Fig. 8f, h). A significant increase in EdU^+^ cells, primarily Tbr2+ intermediate progenitor cells, was observed in the BDNF-increased, FGFR1-activated mice with Numb knockdown, compared with the other two groups (Fig. 8g,i–k).

Notably, light stimulation in aged mice induced antidepressant behavioral phenotypes only when the expression level of Numb was reduced through coadministered shNumb (Supplementary Fig. 10c, d). With no discernible changes in anxiety level and locomotor function (Fig. 8l and Supplementary Fig. 4m–o), immobility time was significantly reduced in FGFR1-activated mice with Numb knockdown, compared with both FGFR1-inactive mice with Numb knockdown and FGFR1-activated mice without Numb knockdown (Fig. 8m).

Overall, these findings suggest that the age-related increase in Numb expression in elderly patients with MDD disrupts the FGFR1–Notch–BDNF axis, hindering the reversal of depressive phenotype; however, selective inhibition of Numb can restore the axis and produce an antidepressant effect.

## Discussion

Numerous studies have documented alterations in the expression of growth factors and their receptors in patients with MDD^16,17,19,36^. However, the precise spatiotemporal dynamics and the interplay among these factors remain unclear, limiting our understanding of MDD pathophysiology. In this study, we identifiy dentate-gyrus-specific upregulation of FGFR1 through comprehensive subregional RNA sequencing and immunohistochemical analysis of the human hippocampus—an approach that has not been thoroughly conducted before. These analyses revealed a novel signaling axis downstream of FGFR1 that regulates key growth factors through Notch signaling activation, playing a pivotal role in AHN, counteracting the depressive phenotype^16^. Notably, we found that Numb is overexpressed in aged individuals with MDD, disrupting the FGFR1–Notch–BDNF axis and contributing to the depressive phenotype.

Previous studies have shown that FGFR1 can form a heterocomplex with 5-HT1A in the hippocampus, particularly in the pyramidal layer of CA1-4 and the dentate gyrus^37^. Activation of the FGFR1-5-HT1A heterocomplex has been associated with antidepressant effects, whereas disturbances in this interaction may lead to a depressive phenotype^37,38^. However, our analysis of human dentate gyrus samples revealed increased expression of FGFR1, 5-HT2A and 5-HT2C receptors, alongside a downregulation of 5-HT1A, differing from the rodent model findings (Supplementary Fig. 11).

Since overexpressed FGFR1 can be activated through homo-oligomerization, we utilized synthetic FGFR1 receptors for inducible activation using an optogenetic approach. Optogenetic platforms enable precise spatiotemporal activation of signaling pathways in a noninvasive manner, providing insights into signaling dynamics at the cellular and organ levels^28^. By integrating the photolyase homology (PHR) domain of cryptochrome 2, which undergoes homo-oligomerization upon blue light exposure, with the cytoplasmic domain of FGFR1, a light-inducible activation module for FGFR1 could be constructed^27,39^. This method enabled systematic analysis of FGFR1 homo-oligomerization signaling, allowing us to track the sequential activation of downstream components in the hippocampal dentate gyrus^25,28^. One such component, Notch signaling, has been implicated in synaptic complexity and survival in mature neurons^40^. However, its precise role in depression has not been well-established. Through the optogenetic dissection of FGFR1 signaling, we identified BDNF as a key downstream effector of the FGFR1-Notch signaling cascade, serving as a protective factor against depression^16^. In addition, the simultaneous increase in FGF2 expression, a ligand for FGFR1, suggests the presence of positive feedback regulation within this axis.

In the human hippocampus, IHC and qRT–PCR analyses of FGFR1 along the rostral-to-caudal axis of the dentate gyrus revealed that FGFR1 expression is predominantly localized to the rostral portion of the dentate gyrus (Supplementary Fig. 12a–c). By contrast, chronic corticosterone administration and CUMS in mice significantly upregulated FGFR1 expression in both the dorsal and ventral dentate gyrus (Supplementary Fig. 13a–f). Although the rostral dentate gyrus in humans has traditionally been considered functionally analogous to the ventral dentate gyrus in rodents, emerging evidence challenges this direct anatomical correspondence, highlighting notable species-specific differences in hippocampal organization, connectivity and function^41–43^. Previous studies have also emphasized the role of the dorsal hippocampus in stress-coping mechanisms, adult neurogenesis and antidepressant responses^44–46^. Consistent with these findings, we observed that BDNF upregulation occurs exclusively in the dorsal dentate gyrus in both CUMS and corticosterone-induced mouse models, despite the global increase in FGFR1 expression across both dorsal and ventral regions (Supplementary Fig. 14a, b). These observations prompted us to further investigate the presence and functional relevance of an FGFR1–Notch–BDNF signaling axis specifically within the dorsal dentate gyrus in relation to depressive phenotypes. Our data demonstrate that conditional deletion of *Fgfr1* in the dorsal dentate gyrus accelerates the onset of depressive-like behaviors, whereas restoration of FGFR1 signaling in this region effectively reverses these effects.

Moreover, we found that activation of the FGFR1–Notch–BDNF axis promotes neural stem cell proliferation within the adult dorsal dentate gyrus, a region where AHN is more prominent than in the ventral dentate gyrus^44,47^, thereby contributing to the observed antidepressant phenotypes. BDNF-mediated AHN is one of the most extensively studied mechanisms underlying the antidepressive effects^26^, yet the precise mechanism by which increased AHN alleviates depressive symptoms remains unclear^48^. Interestingly, rapid-onset antidepressant effects following adult neurogenesis have been reported, which aligns with our findings^49,50^. In our study, behavioral improvements occurred within seven days of initial FGFR1 activation, suggesting that AHN-driven effects precede the full integration of newly generated neurons into the existing neural circuits. Prior research has suggested that increased neuronal activity, as indicated by Egr1 and c-Fos expression, may contribute to these effects^50^. While our study did not directly measure neuronal activity changes, it remains plausible that FGFR1–Notch–BDNF activation-induced AHN contributed to the observed antidepressant phenotype. In addition to AHN, BDNF can exert rapid antidepressive effects independent of neurogenesis, as seen in ketamine-induced antidepressant responses, although the exact molecular mechanisms remain unclear^51,52^. Our findings suggest that FGFR1-induced BDNF upregulation, via Notch activation, may contribute to both direct antidepressant effects and neurogenesis-mediated recovery.

The FGFR1–Notch–BDNF–AHN axis in the dentate gyrus appears to play a protective role against the onset of depressive phenotypes, suggesting that increased FGFR1 expression in MDD is not pathogenic but rather an induced protective response or a defensive mechanism against the full development of the disease. Conditional FGFR1 knockout mice demonstrated that the absence of FGFR1 in the dentate gyrus accelerated the onset of depressive-like behaviors, highlighting its significance during the early stage of the disorder and its compensatory effect on the phenotype. Furthermore, rescue experiments using optoFGFR1 indicate that this axis is essential for the antidepressant effect during the predepressive phase. Dysregulation of this axis by Numb may contribute to the pronounced clinical characteristics observed in elderly patients with MDD, including sudden disease onset and increased suicidal risk^53^. While the exact mechanism underlying Numb upregulation in aged individuals with MDD remains unclear, our study suggests that Numb inhibition reverses BDNF downregulation and restores antidepressant phenotypes, underscoring its therapeutic potential for early-stage MDD in the elderly—a concept that has also been explored in anticancer therapies^54,55^.

This study has several limitations. First, the human postmortem tissue samples used in our study exhibited a skewed sex distribution. Due to prospective collection over a year, obtaining biochemically and molecularly suitable samples while maintaining a balanced male-to-female ratio was challenging. However, prior studies examining FGFR1 expression in human postmortem samples included both males and females and did not report any sex-specific differences in FGFR1 expression^18^, suggesting a minimal impact of sex bias on our results.

Second, we exclusively used male mice due to concerns that hormonal fluctuations, particularly the estrogen cycle, might introduce variability. Previous studies have yielded inconsistent findings regarding sex differences in depressive-like behaviors, with some suggesting that female rodents exhibit more pronounced depressive phenotypes^56,57^. In addition, corticosterone administration, which was used in our study to construct a chemically inducible mouse model, has been shown to increase serum corticosterone levels in females but not in males^58^. Differences in neurotransmitter regulation between sexes further complicate comparisons, warranting future studies to assess sex-specific variations in the FGFR1–Notch–BDNF axis^57^.

To ensure experimental consistency, we utilized both corticosterone-induced and CUMS model^31^. However, other established models, such as the social defeat model^59^ and early-life trauma model^60^ could further validate our findings by replicating specific aspects of MDD pathophysiology.

Lastly, studying depressive phenotypes in aged rodents presents inherent challenges. Our findings align with prior research showing age-dependent variations in corticosterone-induced behavioral responses^61^. While younger mice exhibited no significant changes in anxiety levels, older mice developed increased anxiety after three weeks of corticosterone exposure. Moreover, age-dependent alterations in taste preference were noted, with young mice exhibiting reduced sucrose preference while older mice showed increased preference, potentially linked to metabolic conditions such as diabetes or obesity^62,63^. To minimize confounding effects, we focused on 1-week corticosterone-administered aged mice, a timeframe that mitigates metabolic and systemic biases^64,65^.

In summary, we successfully identified dentate-gyrus-specific upregulation of FGFR1 in the postmortem brains of patients with MDD and uncovered a novel FGFR1–Notch–BDNF axis that promotes neural stem cell proliferation and antidepressant effects. Dysregulation of this axis by Numb, particularly in aged individuals with MDD, contributed to the depressive phenotype. Importantly, inhibiting Numb reversed the axis, suggesting potential therapeutic applications. These results highlight age-dependent variations in FGFR1 expression and provide new insights into the molecular mechanisms underlying depressive disorders.

## Supplementary information

Supplementary Information
