# Supplementary material for: Dysregulation of FGFR1 signaling in the hippocampus facilitates depressive disorder
Source: Exp Mol Med. 2025 Aug 15;57(8):1818–36. doi: 10.1038/s12276-025-01519-9 (PMC12411638; doi:10.1038/s12276-025-01519-9)
Supplement: Supplementary file 1 — Supplementary Information [file 12276_2025_1519_MOESM1_ESM.docx]

**Supplementary Information for**

**Dysregulation of the FGFR1 signaling in hippocampus facilitates depressive disorder**

Jongpil Shin^1^, Hyeonsik Oh^1^, Ji Hye Park^2^, Hyunsik Bae^3,4^, Chan Mo Yang^5^, Minju Lee^2,6,*^, Seokhwi Kim^5,7,*^, Won Do Heo^1,8,*^

^1^Department of Biological Sciences, Korea Advanced Institute of Science and Technology (KAIST), Daejeon, 34141, Republic of Korea.

^2^Forensic Medicine Investigation Division, Seoul Institute, National Forensic Service, Seoul, 08036, Republic of Korea.

^3^Department of Pathology, Severance Hospital, Yonsei University College of Medicine, Seoul, 03722, Republic of Korea

^4^Department of Pathology and Translational Genomics, Samsung Medical Center, Sungkyunkwan University School of Medicine, Seoul, 06351, Republic of Korea.

^5^Department of Biomedical Science, Graduate School of Ajou University, Suwon, 16499, Republic of Korea.

^6^Division of Forensic Medical Examination, Department of Forensic Medicine, National Forensic Service, Wonju, 26460, Republic of Korea.

^7^Department of Pathology, Ajou University School of Medicine, Suwon, 16499, Republic of Korea.

^8^Korea Advanced Institute of Science and Technology Institute for the BioCentury (KIB), Daejeon, 34141, Republic of Korea.

**This file includes:**

Supplementary Materials and Methods

Supplementary Table 1-2

Supplementary Fig. 1-14

**Supplementary Materials and Methods**

**Primary hippocampal neuron culture**

Primary hippocampal neuronal culture was obtained from a pregnant Sprague Dawley (E18) female rat euthanized by CO2. Embryonic hippocampi were dissected from the brain in Hank’s Balanced Salt Solution (HBSS) (Gibco, Cat#14185-052) treated with 10mM HEPES (Gibco, Cat#15630-080). 0.25%Trypsin (Gibco, Cat#15090-046) was treated for 10 minutes at 37℃ then washed with serially diluted Fetal Bovine Serum (FBS) (Gibco, Cat#16140071) in HBSS solution. Trituration was performed with a micropipette and the solution was filtered with a 70-µm cell strainer (BD Falcon, Cat#352350). Neurons were placed on poly-L-lysine (0.1mg/ml) (Merck, Cat#P2636-100mg) pre-coated plates with NM10 medium ((10% horse serum (Gibco, 26050088), 2% Glutamax (Gibco, Cat#35050061), and 1% Antibiotic Antimycotic (Cytiva Hyclone, SV30079.01) solution in neurobasal medium (Gibco, Cat#21103-049)). The cultured neurons were incubated at 37℃ and 5% CO2. The NM10 media was exchanged to a neurobasal medium (Gibco, Cat#21103-049) with B-27 supplement (Gibco, Cat#17504-044) after an hour of incubation. Every third day, 40% of the maintaining medium was replaced with fresh medium.

**Virus production**

The AAV virus production process followed a similar method as described in our previous literature, with minor modifications^1^. Briefly, a three-plasmid co-transfection system was adopted: a plasmid that contains the transgene expressing cassette, a packaging plasmid (pRC-DJ/8), and a helper plasmid (pHelper) were diluted in DPBS (Gibco, Cat#14190-250) at a ratio of 1:1:2 Polyethyleneimine (PEI) was added to the solution to produce a 2:5:1 mixture of DNA and PEI respectively. A light vortexing was performed to aid the compound in mixing uniformly. A 15-minute incubation at room temperature was conducted before the mixture was distributed to 100 pi dishes where HEK 293T cells had been grown to 80% confluency. The cultured medium was completely changed after 4 hours of transfection. After 72 hours of transfection, the cultured supernatant was collected, and a fresh medium was provided. Following the 120 hours of transfection, the cells and cultured supernatant were harvested and centrifuged at 3,300g for 20 minutes.

For lentivirus production, the transfer plasmid, the $\Delta$8.9 plasmid, and the vsvg plasmid were diluted in DPBS (Gibco, Cat#14190-250) at a ratio of 2:1.5:1. PEI was added to the mixture at a ratio of 2.5:1 DNA and PEI respectively. Transfection to HEK293T was performed as described for AAVs. After 72 hours of transfection, the supernatant was collected, centrifuged at 2000 rpm, 4$℃$ for 5 minutes, and filtered with a 0.45µm filter (Sartorius, Cat#S6555-FMOSK). The filtered solution was ultracentrifugated at 25,000 rpm for 90 minutes at 4$℃$. The virus concentrate was later diluted with 70ul of cold DPBS (Gibco, Cat#14190-250).

**In vitro viral transduction**

For the transduction of cultured neurons using AAVs or lentivirus, viral concentrates were dissolved in a neurobasal medium (Gibco, Cat#21103-049) without a B-27 supplement. Half of the maintaining medium was mixed with fresh maintaining medium to produce a conditioned medium. The viral solution was treated to the plate and incubated at 37 $℃$ for one day. All the medium was replaced with a conditioned medium. MOI of 5000 to 8000 was used for transduction.

**Stereotaxic viral injection**

For stereotactic surgery, each mouse was anesthetized with Avertin (2,2,2,-tribromoethanol, 240 mg/kg; Sigma, Cat#T48402). Scalp hair was shaved. A heating pad (Live Cell Instrument) was used to maintain the mouse’s body temperature. An incision was made on the scalp, and craniotomy was performed by drilling the skull (about 0.5 mm in diameter). A viral mixture (volume, 0.5 μl) was infused using a World Precision Instruments (WPI) 33 g blunt NanoFil needle at a rate of 100 nl/min. The stereotactic coordinates used for the dentate gyrus (DG) were AP -2.06, ML 1.2, and DV 1.7. An optic fiber of Ø 200 μm (Doric) was implanted 0.2 mm above the viral injection site after the injection.

**LED stimulation in vitro**

A TouchBright W-96 LED Excitation System (Live Cell Instrument) was utilized for activating optoFGFR1 in cultured neurons. 470nm blue LED was illuminated with an intensity of 5µW/mm^2^ and a duty cycle of 33%. The LED light was illuminated from 0 hours to 24 hours.

**Light stimulation in vivo**

A blue diode 473nm laser (MBL-III-473m; CNI) coupled with optic fiber was used to deliver in vivo light stimulation for activation of optoFGFR1 and RNA-sequencing. The intensity of 5mW/mm^2^ and a duty cycle of 33% were used for light stimulation of the mouse brain. For the depression mice model, light stimulation was delivered through a solid-state LED excitation system using 473nm light (Live Cell Instrument).

**Mouse tissue preparation**

After light stimulation or behavior tests, mice were anesthetized, and their brains were removed from a dark room under a dim red light on ice to minimize light stimulation. The brains were sliced into 1mm sections, and hippocampal DG regions were harvested with a biopsy punch (Kai Medical, Cat#BPP-10F). The samples then froze in liquid nitrogen immediately after punching.

**EdU incorporation and quantification of EdU^+^ cells**

A Click-iT EdU imaging kit (Alexa-647) (Invitrogen, Cat#C10340) was used for detection of EdU incorporation in mitotic cells, following the manufacturer’s protocol. After secondary antibody washing, the click reaction was performed as instructed. The sections were then washed twice with 0.3% Triton-X in DPBS before imaging with a confocal microscope.

The number of EdU^+^ cells in the DG was estimated following the method described by Kim et al^1^. Every sixth 50 μm-thick immunostained hippocampal slice was selected and counted for EdU^+^ cells in the DG. Sampling covered the DG (−1.2 to −3.65 mm from bregma). The total number of EdU^+^ cells was multiplied by six to estimate the total cell count. For calculating the number of cells showing colocalization of EdU with other markers (e.g., Ki-67 or Tbr2), the same strategy was applied to IHC sections.

**RNA isolation, library preparation and sequencing**

Total RNA was isolated using a Trizol reagent (Invitrogen). RNA quality was assessed by Agilent 2100 bioanalyzer using the RNA 6000 Nano Chip (Agilent Technologies, Amstelveen, The Netherlands), and RNA quantification was performed using ND-2000 Spectrophotometer (Thermo Inc., DE, USA).

For control and test RNAs, the construction of the library was performed using QuantSeq 3’ mRNA-Seq Library Prep Kit (Lexogen, Inc., Austria) according to the manufacturer’s instructions. In brief, each 500ng total RNA was prepared and an oligo-dT primer containing an Illumina-compatible sequence at its 5’ end was hybridized to the RNA, and reverse transcription was performed. After degradation of the RNA template, second strand synthesis was initiated by a random primer containing an Illumina-compatible linker sequence at its 5’ end. The double-stranded library was purified by using magnetic beads to remove all reaction components. The library was amplified to add the complete adapter sequences required for cluster generation. The finished library is purified from PCR components. High-throughput sequencing was performed as single-end 75 sequencing using NextSeq 500 (Illumina, Inc., USA).

**Gene Set Enrichment Analysis**

Human hippocampal sub-regional genes from both major depressive disorder (MDD) and normal control were used for GSEA. Gene set analysis was analyzed by GSEA software 4.1.0. The gene set of REACTOME_DOWNSTREAM_SIGNALING_OF_ACTIVATED_FGFR.v2023.1.Hs.grp (M17776) was used for the analysis. GSEA was run for MDD patients versus normal control.

**Conventional animal facility**

Mice were housed in cages with free access to food pellets and water and were kept on a 12-hour light–dark cycle (8 am to 8 pm) at 22 °C and 40% humidity. All behavior experiments were performed during the light phase of the light–dark cycle at the same time of day.

**Reference**

1 Kim, S. *et al.* Dynamic Fas signaling network regulates neural stem cell proliferation and memory enhancement. Sci Adv 6, doi:ARTN eaaz9691

10.1126/sciadv.aaz9691 (2020).

**Supplementary Table 1. Clinical characteristics of human subjects.**

| Group | Case Number | Sex | Age | Duration of MDD | Other medical history | Mode of death | Cause of death | Postmortem interval (PMI) (hour) | RNA integrity number (RIN)  (DG/CA3  /CA2/CA1) |
| --- | --- | --- | --- | --- | --- | --- | --- | --- | --- |
| MDD | 1 | Female | 47 | 7 years | Chronic liver disease | Natural | Chronic alcoholism | 47 | N/A |
|  | 2 | Female | 30 | 3 years | - | Suicide | Drug intoxication | 33 | N/A |
|  | 3* | Male | 55 | 12 years | Diabetes | Suicide | Incised wound | 41 | 2.0/2.0/2.0/2.1 |
|  | 4* | Male | 68 | 2 months | - | Suicide | Hanging | 48 | 2.0/2.1/2.0/2.1 |
|  | 5 | Female | 38 | 2-3 years | - | Suicide | Fall | 31 | N/A |
|  | 6* | Female | 79 | 1 year | Cerebral infraction | Suicide | Hanging | 19 | 2.1/2.1/1.0/2.1 |
| Normal | 1* | Male | 46 | - | Hypertension | Natural | Ischemic heart disease | 49 | 2.0/2.0/2.0/2.0 |
|  | 2* | Male | 25 | - | Hypertension | Natural | Cerebral aneurysmal rupture | 24 | 1.5/2.3/2.1/1.0 |
|  | 3 | Male | 41 | - | Gout | Natural | Pulmonary embolism | 26 | N/A |
|  | 4 | Male | 62 | - | Hypertension | Natural | Aortic dissection | 43 | N/A |
|  | 5* | Male | 58 | - | Hypertension, diabetes, chronic kidney disease, hematologic malignancy | Natural | Acute myocardial infarction | 37 | 2.1/2.2/2.1/2.2 |
|  | 6* | Male | 34 | - | - | Natural | Pneumonia | 38 | 2.0/2.0/2.0/2.1 |
|  | 7 | Male | 38 | - | Heart failure | Natural | Sudden cardiac death | 66 | N/A |
|  | 8* | Male | 53 | - | - | Natural | Acute myocardial infraction | 43 | 2.2/1.0/1.9/1.0 |
|  | 9* | Male | 76 | - | Diabetes, asthma, lung cancer | Natural | Aspergillosis | 28 | 1.0/1.0/3.1/2.4 |

MDD, major depressive disorder, N/A, not available. *Samples subjected to RNA-sequencing analysis.

**Supplementary Table 2. Comparison of clinical characteristics of the study group.**

|  | MDD (N=6) (%) | Normal (N=9) (%) |
| --- | --- | --- |
| Sex  Male  Female | 2 (33.3%)  4 (66.6%) | 9 (100.0%)  0 (0.0%) |
| Age (median, [range]) | 51 [30-79] | 46 [25-76] |
| Postmortem interval (PMI) (median, [range]) (hour) | 37 [19-48] | 38 [24-66] |
| RNA integrity number (RIN)  (median, [range]) | 2.05 [1.0-2.1] | 2.0 [1.0-3.1] |

MDD, major depressive disorder


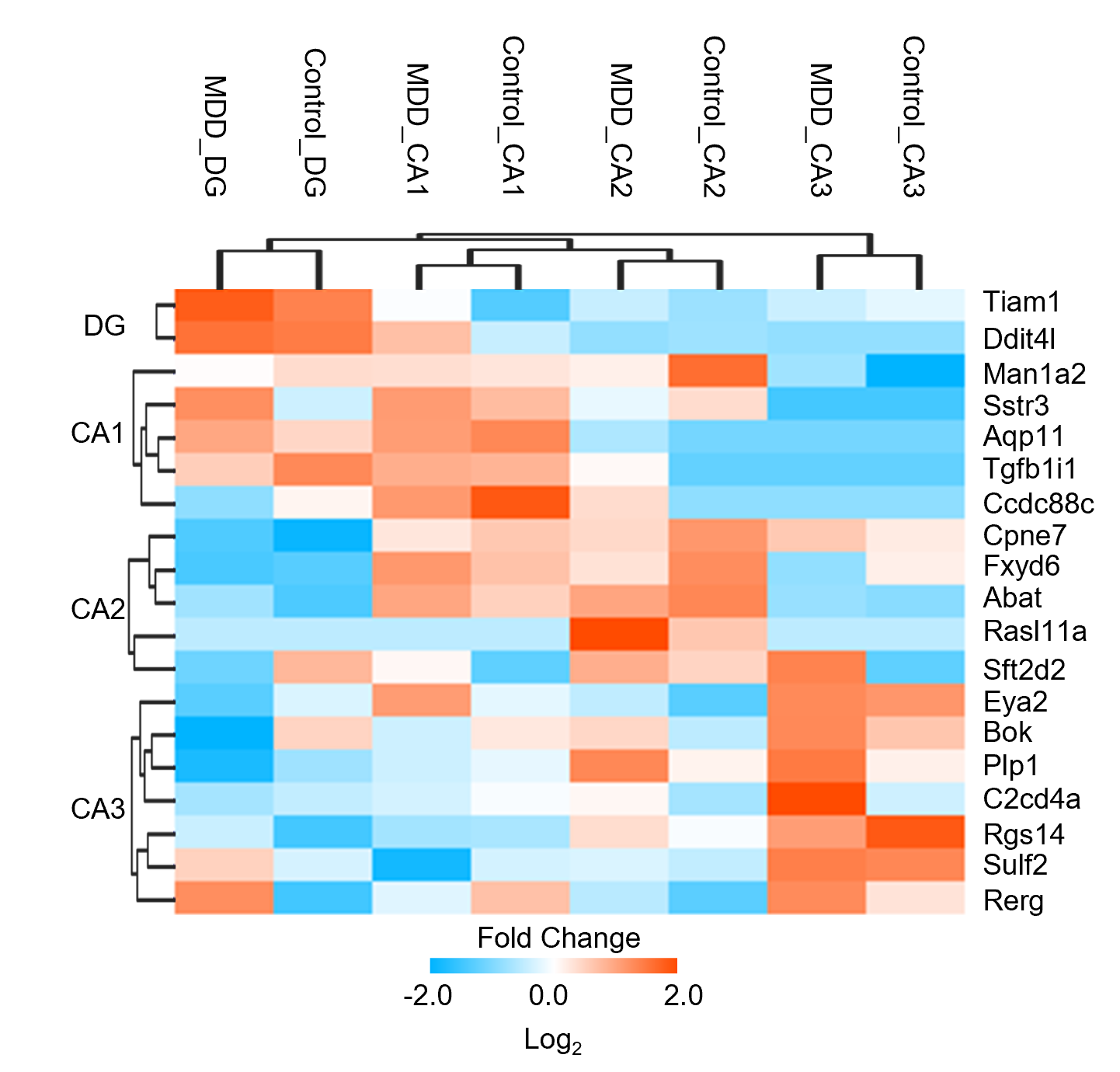


**Supplementary Fig. 1 RNA-sequencing analysis of major depressive disorder patients and normal controls.** Heatmap showing the expression levels of region-specific genes in each hippocampal subregion of major depressive disorder (MDD) patients compared to normal controls. DG, dentate gyrus.


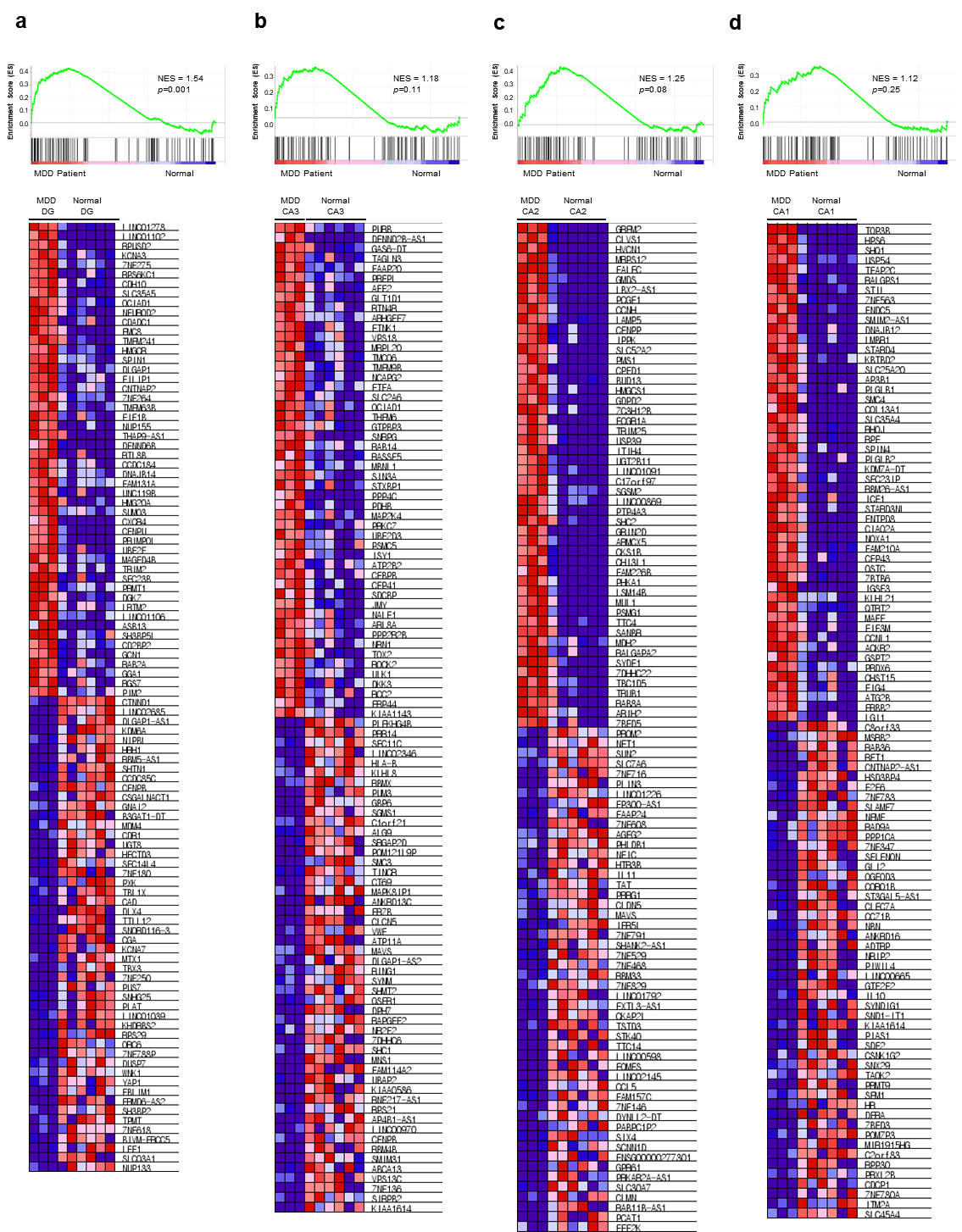


**Supplementary Fig. 2 FGFR1 signaling signature in the dentate gyrus of major depressive disorder patients. a-d** Enrichment plot and heatmap of major depressive disorder (MDD) patients and normal controls in the dentate gyrus (DG) (**a**), CA3 (**b**), CA2 (**c**), and CA1 (**d**) using gene set enrichment analysis (GSEA) of Reactome downstream signaling of activated FGFR (M17776). DG, dentate gyrus.


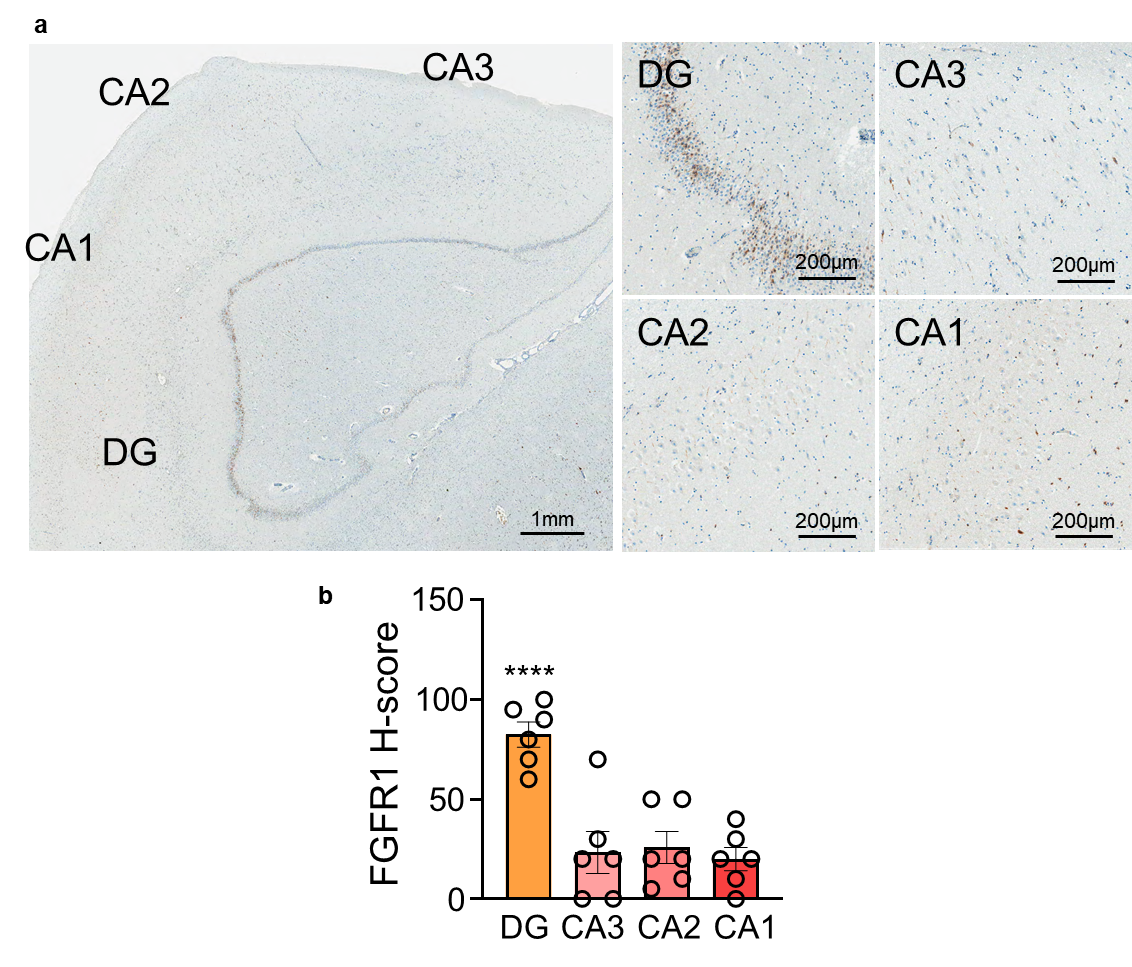


**Supplementary Fig. 3 Regional differences in FGFR1 expression in the hippocampal tissue of major depressive disorder patients. a** Representative immunohistochemical staining (IHC) images of FGFR1 in hippocampal subregions of major depressive disorder (MDD) patients. **b** Quantification of FGFR1 IHC in hippocampal subregions of MDD patients. Data are presented as means ± SEM; *n* = 6 patient samples were included. One-way ANOVA revealed a significant effect of region on FGFR1 expression (*F*(3, 20) = 14.34, *p* < 0.0001). Post hoc comparisons using Tukey’s multiple comparisons test showed significant differences between DG and CA3 (*p* = 0.0002), DG and CA2 (*p* = 0.0003), and DG and CA1 (*p* < 0.0001), but not between CA3 and CA2 (*p* = 0.9959), CA3 and CA1 (*p* = 0.9904), or CA2 and CA1 (*p* = 0.9523). **** *p* < 0.0001. DG, dentate gyrus.


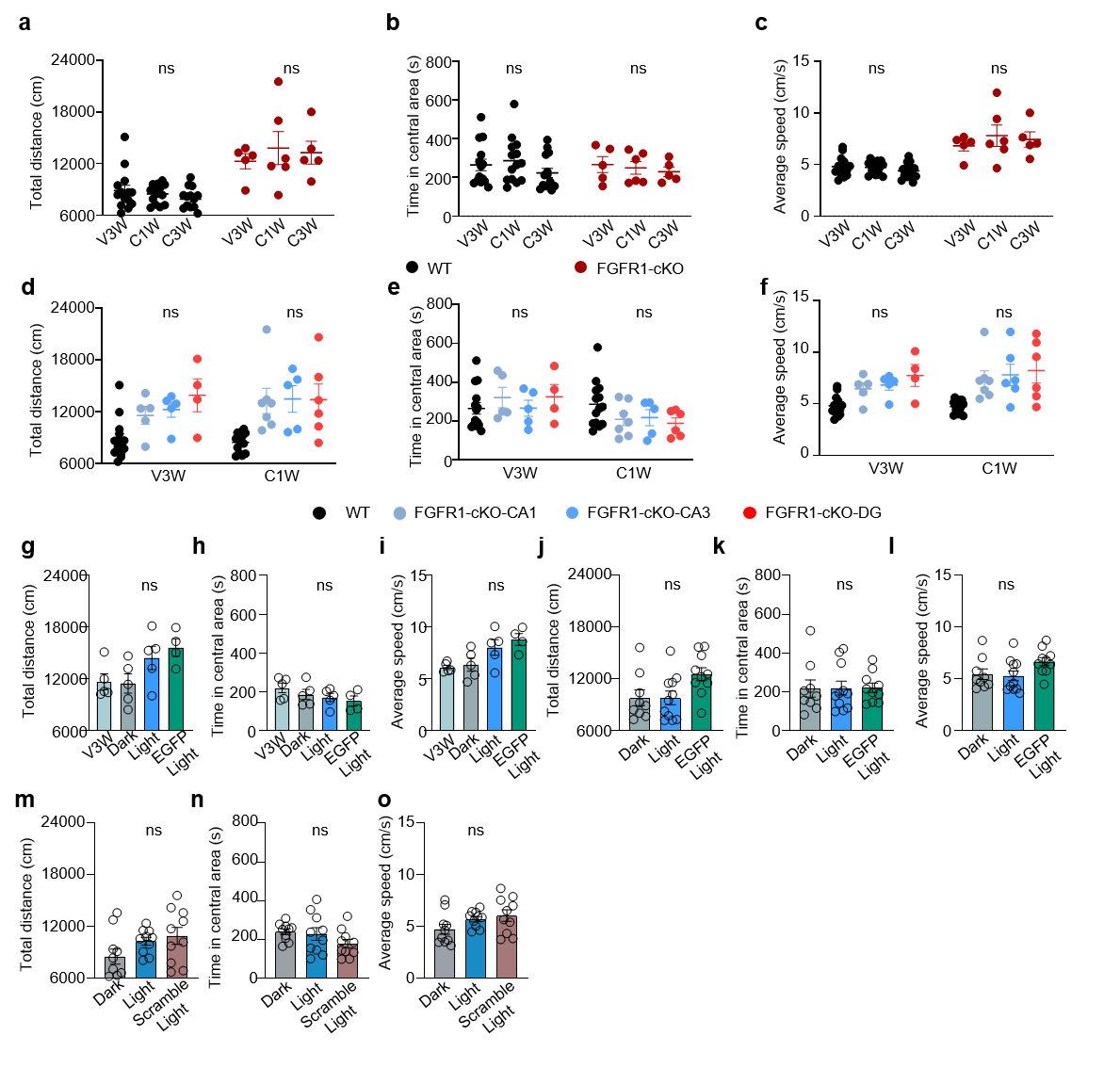


**Supplementary Fig. 4 Analysis of the open field test. a-c** Total distance traveled (**a**), time spent in the central area of the open field chamber (**b**) and average speed (**c**) of wild type (WT) mice and *Fgfr1^flox/flox^* mice. The experimental groups included: administration of vehicle for 3 weeks (V3W); administration of vehicle for 2 weeks followed by corticosterone for 1 week (C1W); and administration of corticosterone for 3 weeks (C3W). WT mice (black) and FGFR1 conditional knockout (FGFR1-cKO) mice, in which a Cre- virus was injected into the hippocampal dentate gyrus of *Fgfr1^flox/flox^* mice (red), were used. Data are presented as means ± SEM; *n* =14 WT mice, *n* = 5 FGFR1-cKO(V3W), *n* = 6 FGFR1-cKO (C1W), and *n* = 5 FGFR1-cKO (C3W) mice were included for each condition. Two-way ANOVA revealed a significant main effect of genotype on total distance traveled (*F*(1, 52) = 49.63, *p* < 0.0001), with no significant effects of corticosterone treatment (*F*(2, 52) = 0.335, *p* = 0.7169) or interaction between genotype and corticosterone treatment (*F*(2, 52) = 0.9425, *p* = 0.3962). Post hoc analysis using Šídák's multiple comparisons test showed no significant differences between conditions within either genotype group. Two-way ANOVA analysis for time spent in the central area revealed no significant main effects of genotype (*F*(1, 52) = 0.1198, *p* = 0.7306), corticosterone treatment (*F*(2, 52) = 0.8310, *p* = 0.4413), or their interaction (*F*(2, 52) = 0.2316, *p* = 0.7941). Post hoc comparisons using Tukey’s test showed no significant differences between treatment conditions within either genotype group. Two-way ANOVA analysis for average speed revealed a significant main effect of genotype (*F*(1, 52) = 61.42, *p* < 0.0001), but no significant main effect of corticosterone treatment (*F*(2, 52) = 0.6815, *p* = 0.5103) or interaction between genotype and corticosterone treatment (*F*(2, 52) = 1.005, *p* = 0.3729). Post hoc comparisons using Šídák’s test showed no significant differences between treatment conditions within either genotype group. ns, not significant. **d-f** Total distance traveled (**d**), time spent in the central area of the open field chamber (**e**) and average speed (**f**) of WT mice and *Fgfr1^flox/flox^* mice. The experimental groups included: WT; FGFR1-cKO mice, in which a Cre- virus was injected into the hippocampal CA1 of *Fgfr1^flox/flox^* mice (FGFR1-cKO-CA1); FGFR1-cKO mice, in which a Cre- virus was injected into the hippocampal CA3 of *Fgfr1^flox/flox^* mice (FGFR1-cKO-CA3); FGFR1-cKO mice, in which a Cre- virus was injected into the hippocampal dentate gyrus of *Fgfr1^flox/flox^* mice (FGFR1-cKO-DG). Data are presented as means ± SEM; *n* =14 WT mice, *n* = 4 (FGFR1-cKO-CA1;V3W), *n* = 5 (FGFR1-cKO-CA1;C1W), *n* = 5 (FGFR1-cKO-CA3;V3W), *n* = 7 (FGFR1-cKO-CA3;C1W), *n* = 5 (FGFR1-cKO-DG;V3W), and *n* = 5 (FGFR1-cKO-DG;C1W) *Fgfr1^flox/flox^* mice were included for each condition. Two-way ANOVA analysis for total distance traveled revealed a significant main effect of hippocampal subregion (*F*(3, 52) = 12.15, *p* < 0.0001), but no significant main effect of corticosterone treatment (*F*(1, 52) = 0.4090, *p* = 0.5253) or interaction between hippocampal subregion and corticosterone treatment (*F*(3, 52) = 0.5414, *p* = 0.6561). Post hoc comparisons using Šídák’s test showed no significant differences between treatment conditions within either V3W or C1W. Two-way ANOVA analysis for central time revealed a significant main effect of corticosterone treatment (*F*(1, 52) = 5.215, *p* = 0.0265), but no significant main effect of hippocampal subregion (*F*(3, 52) = 0.2771, *p* = 0.8417) or interaction between hippocampal subregion and treatment (*F*(3, 52) = 1.920, *p* = 0.1378). Post hoc analysis using Tukey's multiple comparisons test indicated no significant differences between groups at either V3W or C1W. Two-way ANOVA analysis for average speed revealed a significant main effect of hippocampal subregion (*F*(3, 53) = 13.03, *p* < 0.0001), but no significant main effect of corticosterone treatment (*F*(1, 53) = 1.540, *p* = 0.2202) or interaction between hippocampal subregion and corticosterone treatment (*F*(3, 52) = 0.4059, *p* = 0.7494). Post hoc comparisons using Šídák’s test showed no significant differences between treatment conditions within either V3W or C1W. ns, not significant. **g-i** Total distance traveled (**g**), time spent in the central area of the open field chamber (**h**) and average speed (**i**) of *Fgfr1^flox/flox^* mice. The experimental groups included: FGFR1 conditional knockout (FGFR1-cKO) mice with V3W, in which a Cre virus was injected into the hippocampal dentate gyrus of *Fgfr1^flox/flox^* mice (V3W); FGFR1-cKO mice, in which an optoFGFR1 virus was injected into the hippocampal dentate gyrus of *Fgfr1^flox/flox^* mice with C1W under dark room condition (Dark); FGFR1-cKO mice, in which an optoFGFR1 virus was injected into the hippocampal dentate gyrus of *Fgfr1^flox/flox^* mice with C1W and blue LED stimulation for 7 days (Light); FGFR1-EGFP mice, in which a EGFP-virus (control virus) was injected into the hippocampal dentate gyrus of *Fgfr1^flox/flox^* mice followed by C1W and blue LED stimulation for 7 days (EGFP Light). Data are presented as means ± SEM; *n* = 5 (FGFR1-cKO;V3W), *n* = 5 (FGFR1-cKO; Dark), *n* = 5 (FGFR1-cKO; Light), *n* = 4 (FGFR1-EGFP; EGFP Light) *Fgfr1^flox/flox^* mice were included for each condition. One-way ANOVA revealed no significant effect of corticosterone treatment (*F*(3, 15) = 3.191, *p* = 0.0542). Post hoc comparisons using Tukey’s multiple comparisons test showed no significant differences between any group pairs. One-way ANOVA revealed no significant effect of corticosterone treatment on center time (*F*(3, 15) = 1.336, *p* = 0.2998). Post hoc comparisons using Tukey’s multiple comparisons test showed no significant differences between any group pairs. One-way ANOVA revealed no significant effect of corticosterone treatment on average speed (*F*(3, 15) = 2.887, *p* = 0.645). Post hoc comparisons using Tukey’s multiple comparisons test showed no significant differences between any group pairs. ns, not significant. **j-l** Total distance traveled (**j**), time spent in the central area of the open field chamber (**k**), average speed (**l**), of old *Fgfr1^flox/flox^* mice with optoFGFR1 activation. The experimental groups included: old FGFR1-cKO mice, in which an optoFGFR1 virus was injected into the hippocampal dentate gyrus of *Fgfr1^flox/flox^* mice with C1W under dark room condition (Dark); old FGFR1-cKO mice, in which an optoFGFR1 virus was injected into the hippocampal dentate gyrus of *Fgfr1^flox/flox^* mice with C1W and blue LED stimulation for 7 days (Light); old FGFR1-EGFP mice, in which a EGFP-virus (control virus) was injected into the hippocampal dentate gyrus of *Fgfr1^flox/flox^* mice followed by C1W and blue LED stimulation for 7 days (EGFP Light). Data are presented as means ± SEM; *n* = 9 (Dark); *n* = 11 (Light); and *n* = 10 (EGFP Light) old *Fgfr1^flox/flox^* mice were included for each condition. One-way ANOVA revealed a significant effect of corticosterone treatment on distance traveled (*F*(2, 27) = 3.793, *p* = 0.0354). Post hoc comparisons using Tukey’s multiple comparisons test did not show significant differences between group pairs (Dark vs. Light, *p* > 0.9999; Dark vs. EGFP Light, *p* = 0.0706; Light vs. EGFP Light, *p* = 0.0537). One-way ANOVA revealed no significant effect of corticosterone treatment on center time (*F*(2, 27) = 0.0027, *p* = 0.9973). Post hoc comparisons using Tukey’s multiple comparisons test showed no significant differences between group pairs (Dark vs. Light, *p* = 0.9997; Dark vs. EGFP Light, *p* = 0.9972; Light vs. EGFP Light, *p* = 0.9986). One-way ANOVA revealed no significant effect of corticosterone treatment on average speed (*F*(2, 27) = 2.795, *p* = 0.0789). Post hoc comparisons using Tukey’s multiple comparisons test showed no significant differences between group pairs (Dark vs. Light, *p* = 0.9675; Dark vs. EGFP Light, *p* = 0.1728; Light vs. EGFP Light, *p* = 0.0897). ns, not significant. **m-o** Total distance traveled (**m**), time spent in the central area of the open field chamber (**n**), average speed (**o**), old *Fgfr1^flox/flox^* mice with shNumb with optoFGFR1 activation. The experimental groups included: old FGFR1-cKO mice, in which an optoFGFR1 and shNumb virus were injected into the hippocampal dentate gyrus of old *Fgfr1^flox/flox^* mice with C1W under dark room condition (Dark); old FGFR1-cKO mice, in which an optoFGFR1 and shNumb virus were injected into the hippocampal dentate gyrus of old *Fgfr1^flox/flox^* mice with C1W and blue LED stimulation for 7 days (Light); and old FGFR1-cKO mice, in which an optoFGFR1 and shScramble virus were injected into the hippocampal dentate gyrus of old *Fgfr1^flox/flox^* mice with C1W and blue LED stimulation for 7 days (Scramble Light). Data are presented as means ± SEM; *n* =10 mice were included for each condition. One-way ANOVA revealed no significant effect of corticosterone treatment on distance traveled (*F*(2, 27) = 2.375, *p* = 0.1122). Post hoc comparisons using Tukey’s multiple comparisons test showed no significant differences between group pairs (Dark shNumb vs. Light shNumb, *p* = 0.2791; Dark shNumb vs. Light shScramble, *p* = 0.1093; Light shNumb vs. Light shScramble, *p* = 0.8548). One-way ANOVA revealed no significant effect of corticosterone treatment on center time (*F*(2, 27) = 1.804, *p* = 0.1840). Post hoc comparisons using Tukey’s multiple comparisons test showed no significant differences between group pairs (Dark shNumb vs. Light shNumb, *p* = 0.9332; Dark shNumb vs. Light shScramble, *p* = 0.1909; Light shNumb vs. Light shScramble, *p* = 0.3359). One-way ANOVA revealed no significant effect of corticosterone treatment on average speed (*F*(2, 27) = 2.359, *p* = 0.1137). Post hoc comparisons using Tukey’s multiple comparisons test showed no significant differences between group pairs (Dark shNumb vs. Light shNumb, *p* = 0.282; Dark shNumb vs. Light shScramble, *p* = 0.1107; Light shNumb vs. Light shScramble, *p* = 0.8547). ns, not significant.


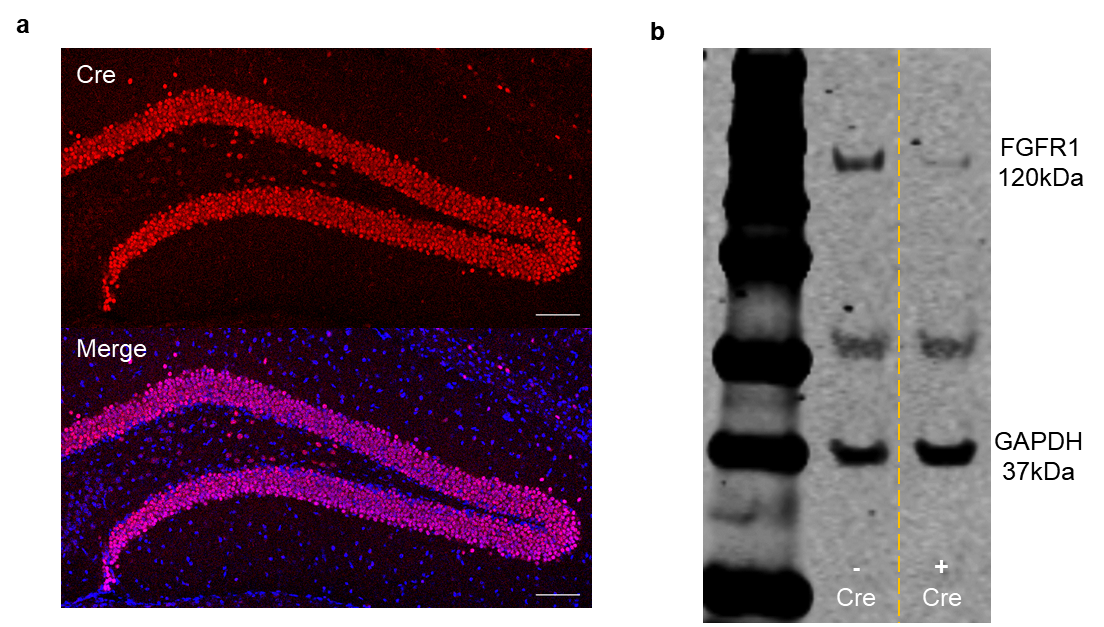


**Supplementary Fig. 5 Conditional knockout of FGFR1 in mouse hippocampal regions. a** Representative immunohistochemical staining images of Cre in the dentate gyrus (DG) of *Fgfr1^flox/flox^* mice. Scale bar, 100 μm. **b** Western blot analysis of FGFR1 and GAPDH following Cre viral injection in the DG of *Fgfr1^flox/flox^* mice. DG, dentate gyrus.


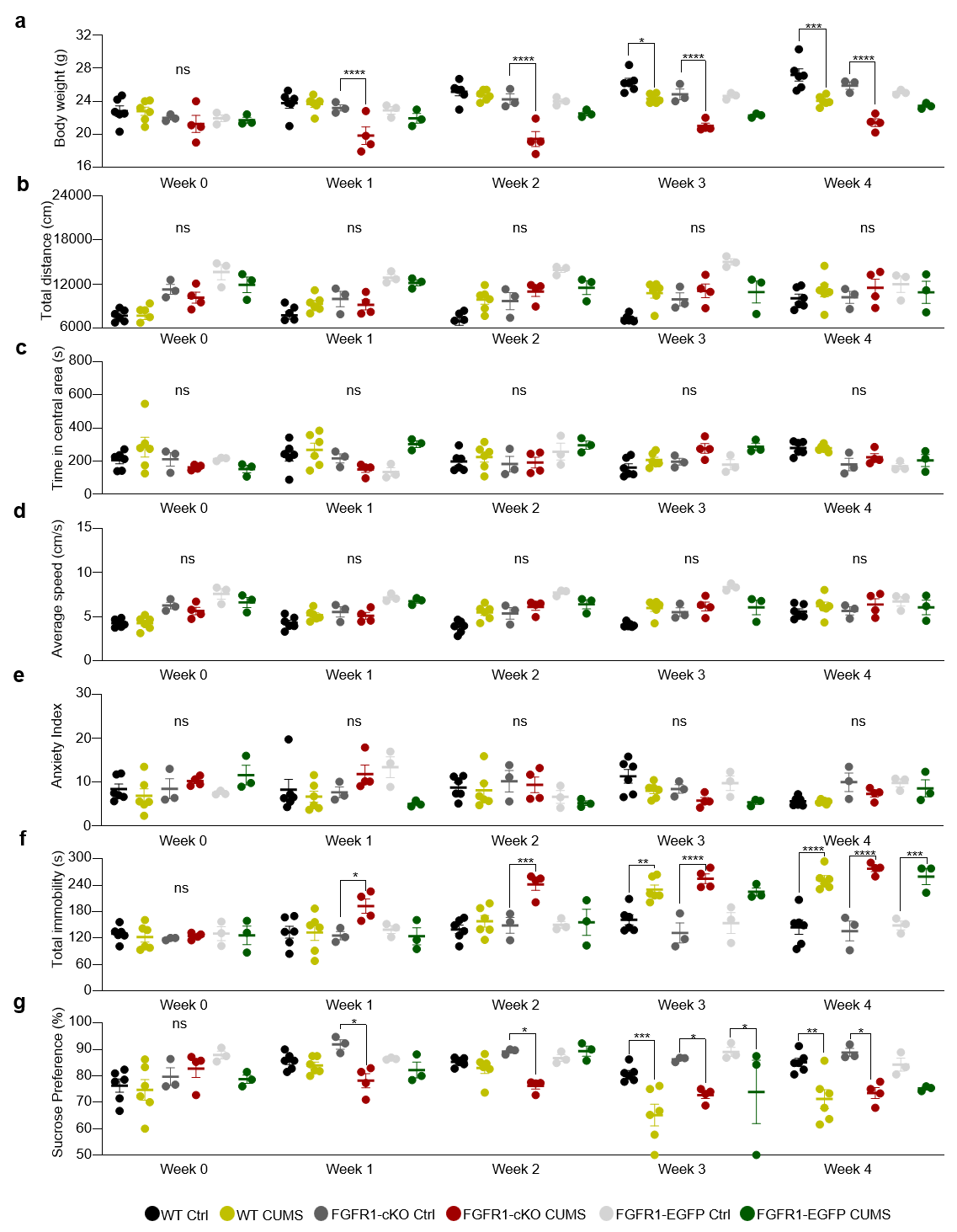


**Supplementary Fig. 6 Analysis of depressive-like behaviors under the chronic unpredictable mild stress model. a** Body weight of mouse subjected to chronic unpredictable mild stress (CUMS). The experimental conditions included: (1) CUMS; (2) Control (Ctrl), consisting of mice not subjected to stress. Three types of mice were used: (1) wild type (WT) mice; (2) FGFR1 conditional knockout (FGFR1-cKO) mice, in which a Cre-expressing virus was injected into the hippocampal dentate gyrus of *Fgfr1^flox/flox^* mice; and (3) FGFR1-EGFP mice, in which a control EGFP-expressing virus (non-Cre) was injected into the hippocampal dentate gyrus of *Fgfr1^flox/flox^* mice. Data are presented as means ± SEM; *n* = 6 WT mice, *n* = 3 FGFR1-cKO Ctrl mice, *n* = 4 FGFR1-cKO-CUMS mice, *n* = 3 FGFR1-EGFP Ctrl mice, and *n* = 3 FGFR1-EGFP CUMS mice were included for each condition. Two-way ANOVA revealed significant main effects of CUMS weeks (*F*(4, 75) = 11.33, *p* < 0.0001) and genotype (*F*(3, 75) = 52.80, *p* < 0.0001), as well as a significant interaction between CUMS weeks and genotype (*F*(12, 75) = 2.748, *p* = 0.0038). Post hoc comparisons using Tukey’s multiple comparisons test showed that FGFR1-cKO CUMS mice exhibited significantly reduced body weight compared to WT CTRL, WT CUMS, and FGFR1-cKO Ctrl mice from Week 1 onward (*p* < 0.01 to *p* < 0.0001). WT CUMS mice also showed significantly lower body weight than WT CTRL mice at Week 3 (*p* = 0.0358) and Week 4 (*p* = 0.0003). ns, not significant; **p* <0.05; ****p* <0.001; *****p* < 0.0001. **b** Total distance traveled in open field test of mouse subjected to CUMS. Data are presented as means ± SEM; *n* = 6 WT mice, *n* = 3 FGFR1-cKO Ctrl mice, *n* = 4 FGFR1-cKO-CUMS mice, *n* = 3 FGFR1-EGFP Ctrl mice, and *n* = 3 FGFR1-EGFP CUMS mice were included for each condition. Two-way ANOVA revealed significant main effects of CUMS weeks (*F*(4, 95) = 29.91, *p* < 0.0001) and genotype (*F*(5, 95) = 2.028, *p* = 0.0123), as well as a significant interaction between CUMS weeks and genotype (*F*(20, 95) = 2.028, *p* = 0.0123). Post hoc comparisons using Tukey’s multiple comparisons test showed no significant differences were observed for the other genotypes across the weeks. ns, not significant. **c** Time spent in the central area of the open field chamber mouse subjected to CUMS. Data are presented as means ± SEM; *n* = 6 WT mice, *n* = 3 FGFR1-cKO Ctrl mice, *n* = 4 FGFR1-cKO-CUMS mice, *n* = 3 FGFR1-EGFP Ctrl mice, and *n* = 3 FGFR1-EGFP CUMS mice were included for each condition. Two-way ANOVA revealed a significant interaction between CUMS weeks and genotype (*F*(20, 95) = 2.253, *p* = 0.0047), as well as significant main effects of genotype (*F*(5, 95) = 3.441, *p* = 0.0067) but not CUMS weeks (*F*(4, 95) = 0.3275, *p* = 0.8589). Post hoc comparisons using Tukey’s multiple comparisons test showed no significant differences were observed for the other genotypes across the weeks. ns, not significant. **d** Average speed of mouse subjected to CUMS. Data are presented as means ± SEM; *n* = 6 WT mice, *n* = 3 FGFR1-cKO Ctrl mice, *n* = 4 FGFR1-cKO-CUMS mice, *n* = 3 FGFR1-EGFP Ctrl mice, and *n* = 3 FGFR1-EGFP CUMS mice were included for each condition. Two-way ANOVA revealed no significant effects of CUMS weeks (*F*(4, 95) = 0.9889, *p* = 0.4175) and genotype (*F*(5, 95) = 30.30, *p* < 0.0001), as well as a significant interaction between CUMS weeks and genotype (*F*(20, 95) = 1.997, *p* = 0.0140). Post hoc comparisons using Tukey’s multiple comparisons test showed no significant differences were observed for the other genotypes across the weeks. ns, not significant. **e** Anxiety index of mice subjected to CUMS. Data are presented as means ± SEM; *n* = 6 WT mice, *n* = 3 FGFR1-cKO Ctrl mice, *n* = 4 FGFR1-cKO-CUMS mice, *n* = 3 FGFR1-EGFP Ctrl mice, and *n* = 3 FGFR1-EGFP CUMS mice were included for each condition. Two-way ANOVA revealed no significant effects of CUMS weeks (*F*(4, 95) = 0.5606, *p* = 0.6918) and genotype (*F*(5, 95) = 2.127, *p* = 0.0688), as well as a significant interaction between CUMS weeks and genotype (*F*(20, 95) = 2.063, *p* = 0.0106). Post hoc comparisons using Tukey’s multiple comparisons test showed no significant differences were observed for the other genotypes across the weeks. ns, not significant. **f** Total immobility time in tail suspension test of mouse subjected to CUMS. Data are presented as means ± SEM; *n* = 6 WT mice, *n* = 3 FGFR1-cKO Ctrl mice, *n* = 4 FGFR1-cKO-CUMS mice, *n* = 3 FGFR1-EGFP Ctrl mice, and *n* = 3 FGFR1-EGFP CUMS mice were included for each condition. Two-way ANOVA revealed significant main effects of CUMS weeks (*F*(4, 95) = 28.92, *p* < 0.0001) and genotype (*F*(5, 95) = 24.13, *p* < 0.0001), as well as a significant interaction between CUMS weeks and genotype (*F*(20, 95) = 4.453, *p <* 0.0001). Post hoc comparisons using Tukey’s multiple comparisons test showed that FGFR1-cKO-CUMS mice displayed significant behavioral changes compared to FGFR1-cKO Ctrl mice, with significant differences from Week 0 to Weeks 1, 2, 3, and 4 (*p* < 0.05 to *p* < 0.0001). WT CUMS mice exhibited significant behavioral changes compared to WT Ctrl, with significant differences observed at Weeks 3 and 4 (*p* < 0.0001). WT Ctrl and FGFR1-cKO Ctrl mice showed no significant differences across the time points (*p* > 0.05). **g** Sucrose preference in mouse subjected to CUMS. Data are presented as means ± SEM; *n* = 6 WT mice, *n* = 3 FGFR1-cKO Ctrl mice, *n* = 4 FGFR1-cKO-CUMS mice, *n* = 3 FGFR1-EGFP Ctrl mice, and *n* = 3 FGFR1-EGFP CUMS mice were included for each condition. Two-way ANOVA revealed significant main effects of CUMS weeks (*F*(4, 95) = 6.493, *p* = 0.0001) and genotype (*F*(5, 95) = 13.91, *p* < 0.0001), as well as a significant interaction between CUMS weeks and genotype (*F*(20, 95) = 2.381, *p* = 0.0027). Post hoc comparisons using Tukey’s multiple comparisons test showed that FGFR1-cKO CUMS mice displayed significant behavioral changes compared to FGFR1-cKO Ctrl mice, with significant differences from Week 0 to Weeks 1, 2, 3, and 4 (*p* = 0.0146 to *p* = 0.0028). WT CUMS mice exhibited significant behavioral changes compared to WT Ctrl, with significant differences observed at Weeks 3 and 4 (*p* = 0.0002 to *p* < 0.0001). WT Ctrl and FGFR1-cKO Ctrl mice showed no significant differences across the time points (p > 0.05). ns, not significant; * *p* <0.05; ** *p* <0.01; *** *p* <0.001; **** *p* < 0.0001.


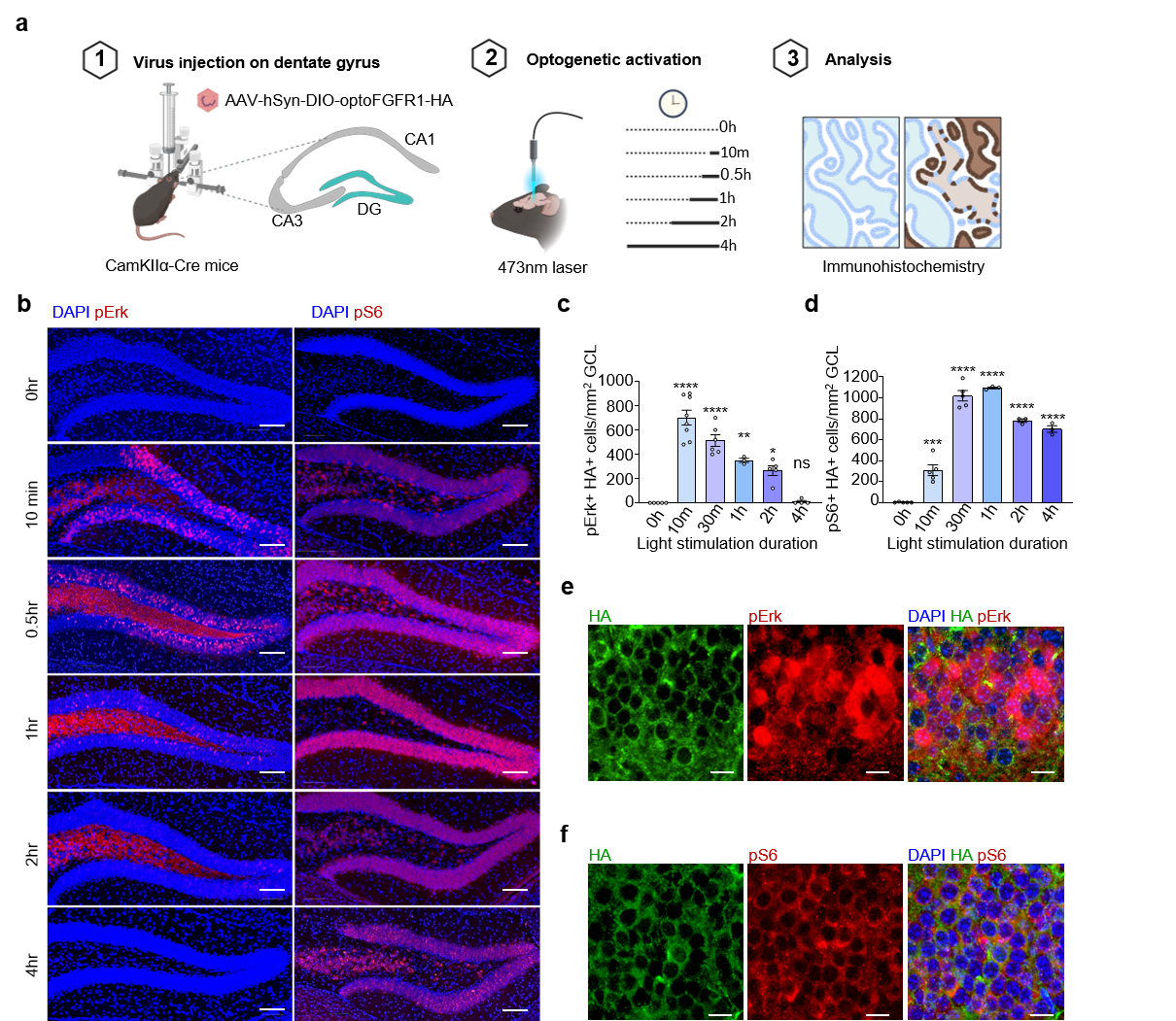


**Supplementary Fig. 7 Optogenetic activation of FGFR1 in neurons of the hippocampal dentate gyrus induces known FGFR1 downstream signaling components. a** Schematic representation and timeline showing the viral injection and blue light stimulation on CaMKII$\alpha$-Cre mice. AAV-hSyn-DIO-optoFGFR1-HA virus was injected in hippocampal DG. **b** Representative images of changes in the levels of pErk and pS6 in the optoFGFR1-transduced dentate gyrus as the duration of illumination increased. Scale bar, 100 μm. **c** Quantification of the pErk-positive cells in **b**. Data are presented as means ± SEM. The number of mice included for each condition was follows: *n* = 5 for 0 hour light stimulation, *n* = 8 for 10 minutes light stimulation, *n* = 6 for 30 minutes light stimulation, *n* = 3 for 1 hour light stimulation, *n* = 5 for 2 hours light stimulation, and *n* = 4 for 4 hours light stimulation. One-way ANOVA revealed a significant effect of optoFGFR1 activation (*F*(5, 25) = 36.36, *p* < 0.0001). Post hoc comparisons using Tukey’s multiple comparisons test showed significant differences between several group pairs (0 vs. 10min, *p* < 0.0001; 0 vs. 0.5 hr, *p* < 0.0001; 0 vs. 1 hr, *p* = 0.0028; 0 vs. 2 hr, *p* = 0.0101). ns, not significant; **p* <0.05; ***p* <0.01; *****p* < 0.0001. **d** Quantification of the pS6-positive cells in **b**. Data are presented as means ± SEM. The number of mice included for each condition was follows: *n* = 5 for 0 hour light stimulation, *n* = 5 for 10 minutes light stimulation, *n* = 5 for 30 minutes light stimulation, *n* = 3 for 1 hour light stimulation, *n* = 3 for 2 hours light stimulation, and *n* = 3 for 4 hours light stimulation. One-way ANOVA revealed a significant effect of optoFGFR1 activation (*F*(5, 17) = 112.1, *p* < 0.0001). Post hoc comparisons using Tukey’s multiple comparisons test showed significant differences between several group pairs (0 vs. 10 min, p = 0.0002; 0 vs. 0.5 hr, p < 0.0001; 0 vs. 1 hr, p < 0.0001; 0 vs. 2 hr, p < 0.0001; 0 vs. 4 hr, p < 0.0001). ns, not significant; ****p* <0.001; *****p* < 0.0001. **e** Representative images showing the co-localization of HA+ cells and pERK+ cells. Scale bar, 20 μm. **f**, Representative images showing the co-localization of HA+ cells and pS6+ cells. Scale bar, 20 μm.


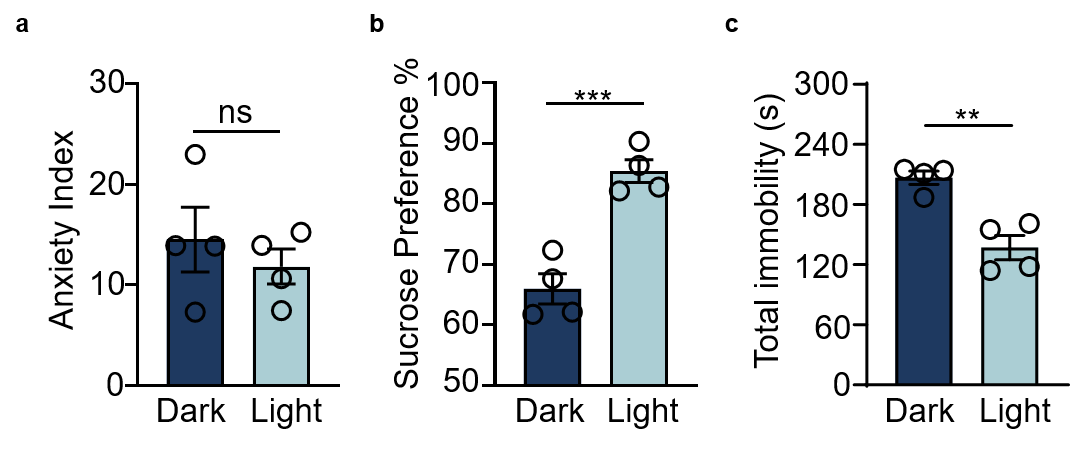


**Supplementary Fig. 8 Optogenetic rescue of depressive-like behaviors in *Fgfr1^flox/flox^*** **mice via adult hippocampal neurogenesis.** **a-c** Anxiety index (**a**), and sucrose preference (**b**), and total immobility time in tail suspension test (**c**) in *Fgfr1^flox/flox^* mice. Data are presented as means ± SEM; *n* = 4 mice in each condition. For anxiety index, an unpaired two-tailed *t*-test revealed no significant difference between the light and dark group (*t*(6) = 0.7351, *p* = 0.49). For the sucrose preference, an unpaired two-tailed *t*-test revealed a significant increase in the light group compared to the dark group (*t*(6) = 6.184, *p* = 0.0008). For the total immobility, an unpaired two-tailed *t*-test revealed a significant decrease in the light group compared to the dark group (*t*(6) = 5.018, *p* = 0.0024). ns, not significant; ***p* < 0.01; ****p* < 0.001.


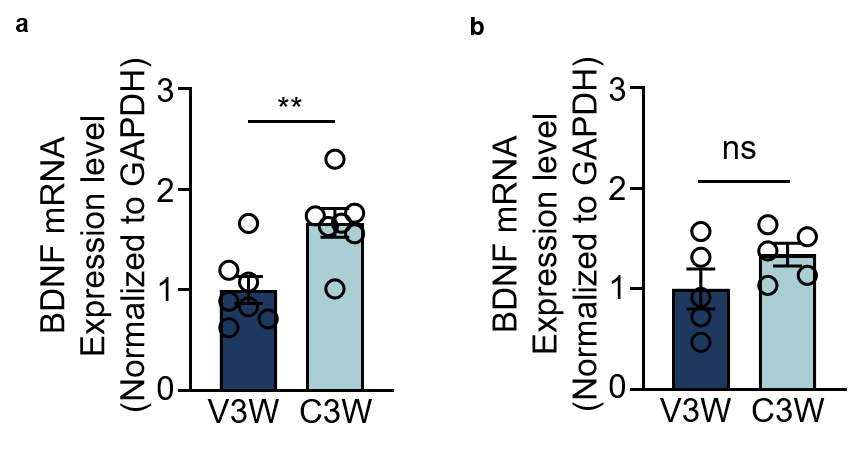


**Supplementary Fig. 9 Age-dependent BDNF expression in a mouse depression model.**

**a** qRT-PCR results for BDNF in corticosterone-induced depression model of young wild type (WT) mice. V3W, vehicle administration of 3 weeks; C3W, corticosterone administration of 3 weeks. Data are presented as means ± SEM; *n* = 7 per each condition. An unpaired two-tailed *t*-test revealed a significant increase in the young C3W compared to the V3W (*t*(12) = 3.416, *p* = 0.0051). ***p* < 0.01. **b** qRT-PCR results for BDNF in corticosterone-induced depression model of old WT mice. V3W, vehicle administration of 3 weeks; C3W, corticosterone administration of 3 weeks. Data are presented as means ± SEM; *n* = 5 per each condition. An unpaired two-tail t-test was used for statistical analysis. ns, not significant. An unpaired two-tailed *t*-test revealed no significant difference between the old C3W and V3W groups (*t*(8) = 1.494, *p* = 0.1735). ns, not significant.


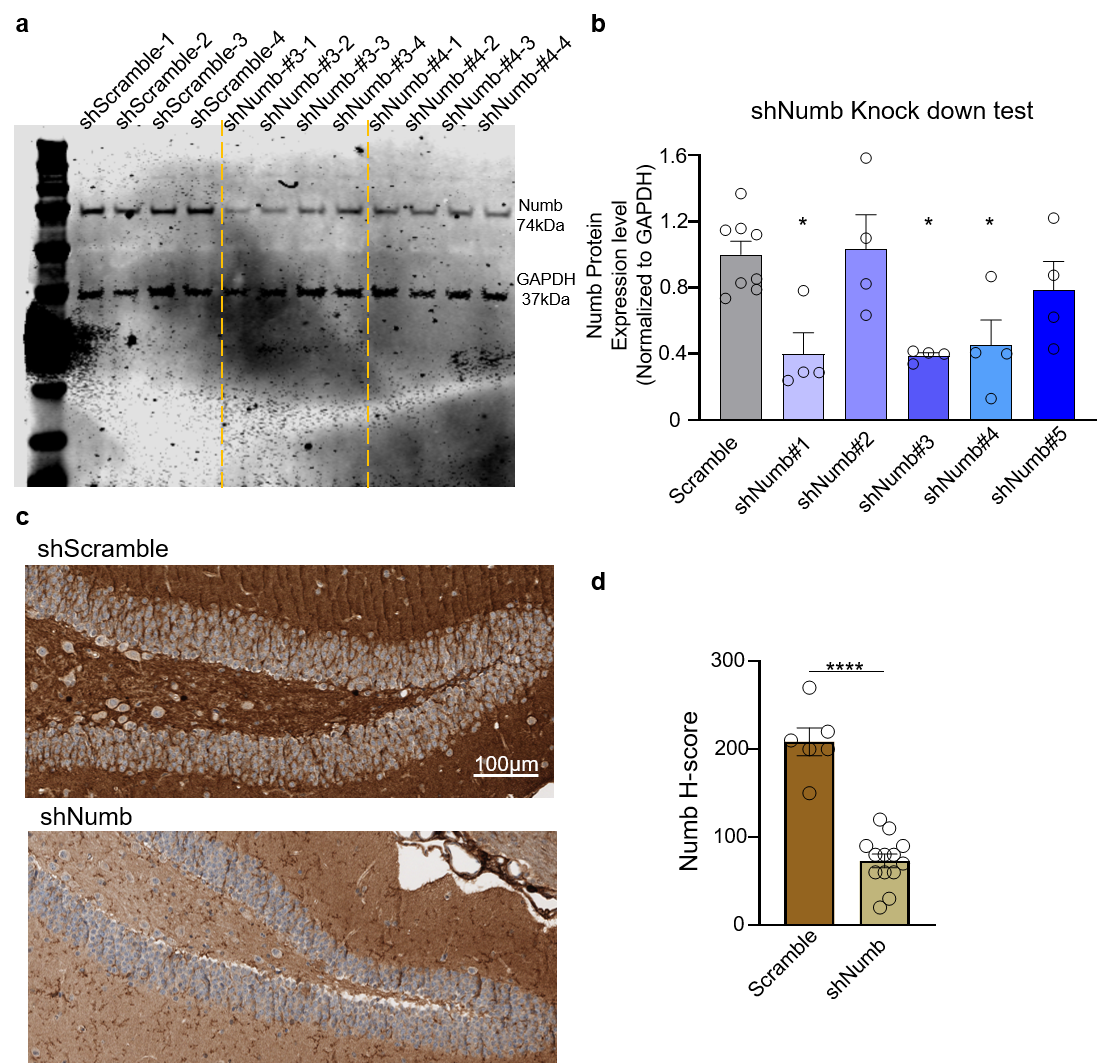


**Supplementary Fig. 10 Knockdown of Numb. a** Western Blot for Numb and GAPDH of shScramble- and shNumb-injected wild type (WT) mice hippocampus. **b** Quantification of Numb to GAPDH from (**a**) showing significant decreased protein level compared to shScramble. Data are represented as means ± SEM; *n* = 8 for shScramble, *n* = 4 for shNumb#1, *n* = 4 for shNumb#2, *n* = 4 for shNumb#3, *n* = 4 for shNumb#4, and *n*= 4 for shNumb#5 injected WT mice were included for each condition. One-way ANOVA revealed a significant effect of treatment on the shNumb efficiency (*F*(5, 22) = 5.617, *p* = 0.0018). Post hoc Tukey's multiple comparisons test showed significant differences between several group pairs, including: Scramble vs. shNumb#1 (*p* = 0.0207), Scramble vs. shNumb#3 (*p* = 0.0182), and Scramble vs. shNumb#4 (*p* = 0.0401). Other comparisons did not show significant differences (p > 0.05). **p* <0.05. **c** Representative immunohistochemical staining images of Numb in shScramble- and shNumb-injected old *Fgfr1^flox/flox^* mice dentate gyrus (DG). **d** Quantification of Numb IHC in shScramble- and shNumb-injected old *Fgfr1^flox/flox^* mice DG. Data are represented as mean ± SEM; n =6 and 13 mice were included for each condition. The Mann-Whitney U test revealed a significant difference between the shScramble and shNumb groups (*U* = 0, *p* < 0.0001). The median of the shScramble group was 205.0 (*n* = 6), while the median of the shNumb group was 80.00 (*n* = 13), showing a significant difference of -125. The Hodges-Lehmann estimate of the difference between medians was -130. *****p* <0.0001.


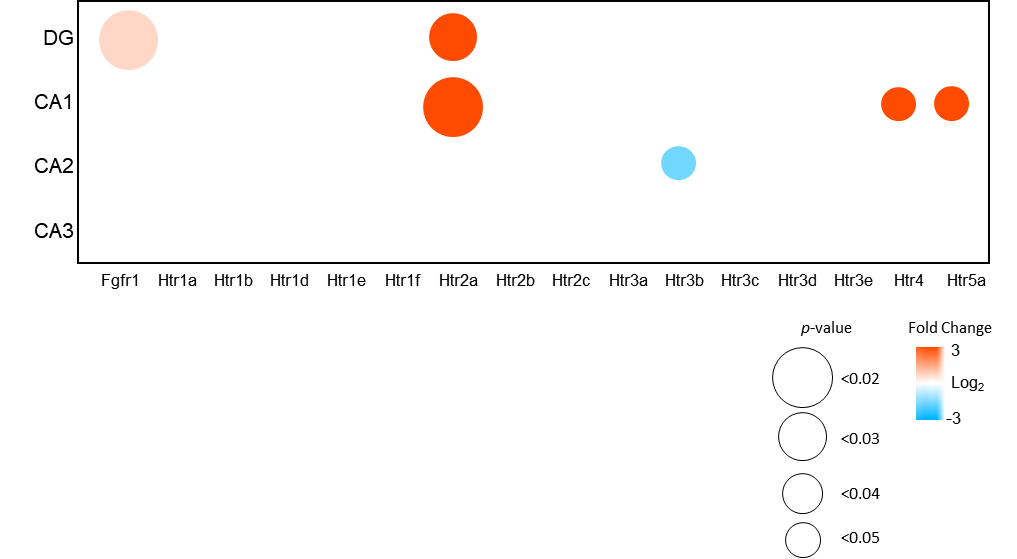


**Supplementary Fig. 11 Dot plot showing the expression levels of 5-HT-related genes in each hippocampal subregion of major depressive disorder patients compared to normal controls.**


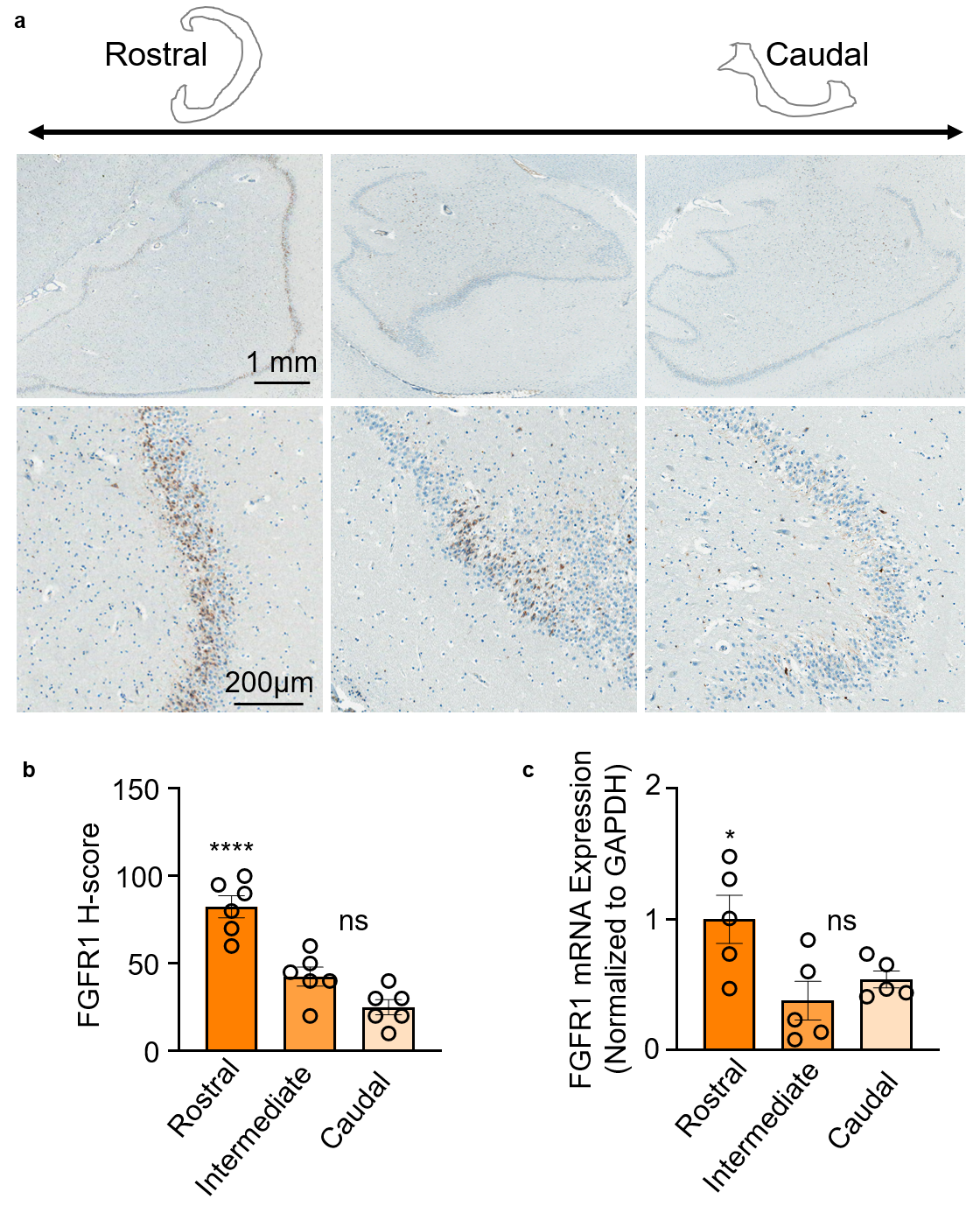


**Supplementary Fig. 12 Differences in FGFR1 expression along rostral-to-caudal axis in the dentate gyrus of major depressive disorder patients. a** Representative immunohistochemical staining (IHC) images of FGFR1 in dentate gyrus (DG) of major depressive disorder (MDD) patients, along rostral-to-caudal axis. **b** Quantification of FGFR1 IHC in DG of MDD patients along rostral-to-caudal axis. Data are presented as means ± SEM; *n* = 6 patient samples were included. One-way ANOVA showed a significant effect of rostro-caudal position on FGFR1 expression (*F*(2, 15) = 29.79, *p* < 0.0001). Post hoc comparisons using Tukey’s multiple comparisons test showed significant differences between the rostral and intermediate regions (*p* = 0.0003), and between the rostral and caudal regions (*p* < 0.0001). There was no significant difference between intermediate and caudal regions (*p* = 0.088). ns not significant; *****p* < 0.0001. **c** FGFR1 qRT-PCR results from DG of MDD patients along rostral-to-caudal axis. Data are presented as means ± SEM; *n* = 5 patient samples were included. One-way ANOVA showed a significant effect of rostro-caudal position on FGFR1 expression (*F*(2, 12) = 5.2218, *p* = 0.0234). Post hoc comparisons using Tukey’s multiple comparisons test showed significant differences between the rostral and intermediate regions (*p* = 0.0226). There was no significant difference between intermediate and caudal regions (*p* = 0.7008). ns not significant; **p* < 0.05.

**
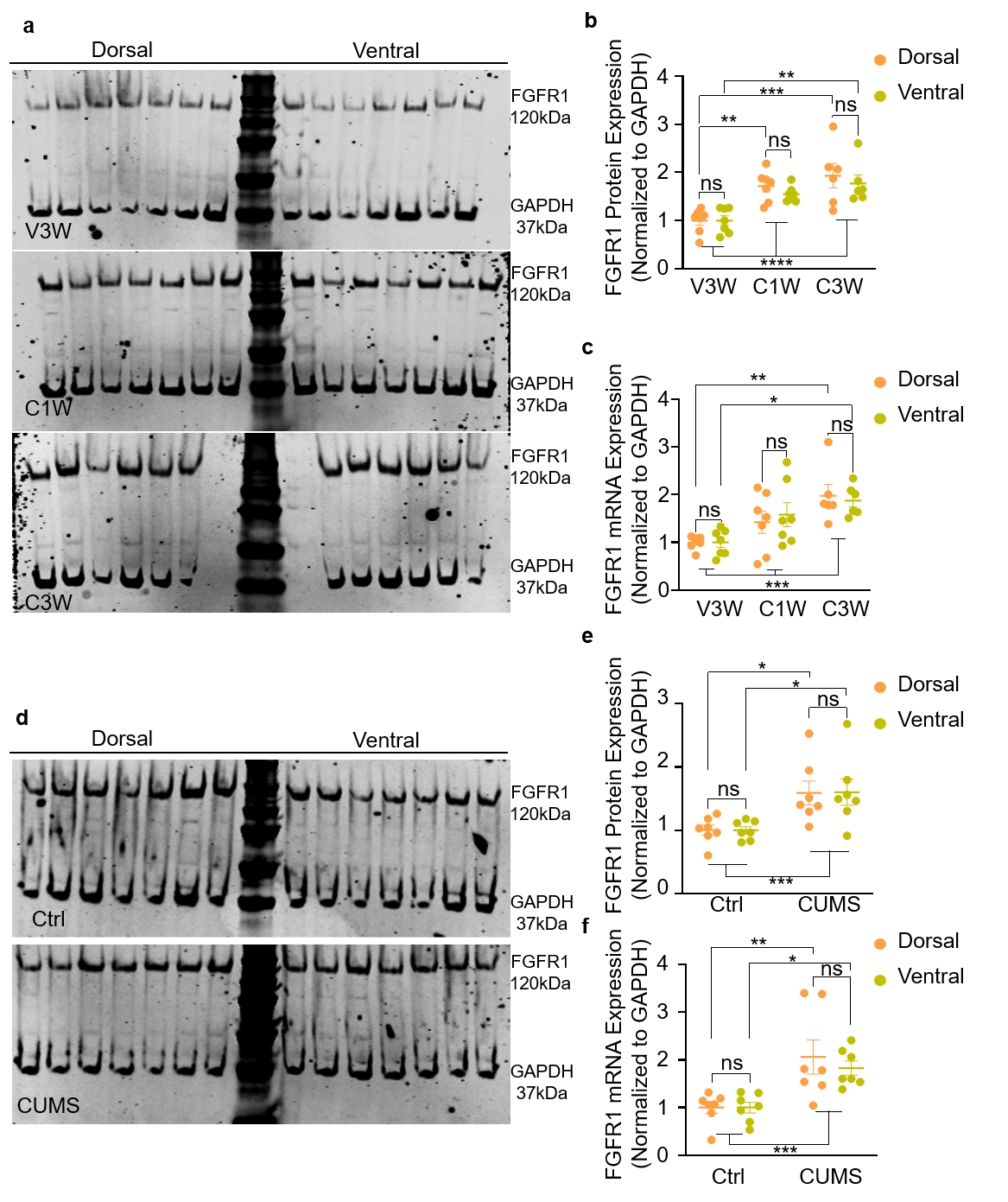
**

**Supplementary Fig. 13 Mouse depression models induces upregulation of FGFR1 in both dorsal and ventral dentate gyrus.**

**a** Western Blot for FGFR1 and GAPDH in the dorsal and ventral hippocampus of WT mice in a corticosterone induced depression model. V3W(top); C1W (middle); C3W (bottom). **b** Quantification of FGFR1to GAPDH from (**a**). Two-way ANOVA revealed a significant main effect of corticosterone treatment (*F*(2, 34) = 20.19, *p* < 0.0001), but no significant effects of region (*F*(1, 34) = 0.9144, *p* = 0.3457) or interaction between treatment and region (*F*(2, 34) = 0.2368, *p* = 0.79904). Post hoc comparisons using Tukey’s multiple comparisons test showed significant differences between several group pairs (WT V3W Dorsal vs C1W Dorsal, *p* = 0.0084; WT V3W Dorsal vs C3W Dorsal, *p* = 0.0006; WT V3W Ventral vs C3W Ventral, p = 0.0058). No significant differences were observed between dorsal and ventral regions within each group. ns, not significant; ***p* < 0.01; *** *p* < 0.001; **** *p* < 0.0001. **c** FGFR1 qRT-PCR results in the dorsal and ventral hippocampus of WT mice in a corticosterone induced depression model. Two-way ANOVA revealed a significant main effect of corticosterone treatment (*F*(2, 34) = 12.20, *p* = 0.0001), but no significant effects of region (*F*(1, 34) = 0.01457, *p* = 0.9046) or interaction between treatment and region (*F*(2, 34) = 0.2463, *p* = 0.7831). Post hoc comparisons using Tukey’s multiple comparisons test showed significant differences between several group pairs (WT V3W Dorsal vs C3W Dorsal, *p* = 0.0098; WT V3W Ventral vs C3W Ventral, p = 0.0264). No significant differences were observed between dorsal and ventral regions within each group. ns, not significant; **p* < 0.05; ***p* < 0.01; *** *p* < 0.001. **d** Western Blot for FGFR1 and GAPDH in the dorsal and ventral hippocampus of WT mice in CUMS model. Ctrl(top); CUMS (bottom). **e** Quantification of FGFR1to GAPDH from (**d**). Two-way ANOVA revealed a significant main effect of CUMS (*F*(1, 24) = 16.19, *p* = 0.0005), but no significant effects of region (*F*(1, 24) = 0.001840, *p* = 0.9661) or interaction between CUMS and region (*F*(1, 24) = 0.001840, *p* = 0.9661). Post hoc comparisons using Tukey’s multiple comparisons test showed significant differences between two group pairs (WT Ctrl Dorsal vs CUMS Dorsal, *p* = 0.00443; WT Ctrl Ventral vs CUMS Ventral, p = 0.0388). No significant differences were observed between dorsal and ventral regions within each group. ns, not significant; **p* < 0.05; *** *p* < 0.001.**f** FGFR1 qRT-PCR results in the dorsal and ventral hippocampus of WT mice in CUMS model. Two-way ANOVA revealed a significant main effect of CUMS (*F*(1, 24) = 20.25, *p* = 0.0001), but no significant effects of region (*F*(1, 24) = 0.3097, *p* = 0.5830) or interaction between CUMS and region (*F*(1, 24) = 0.3097, *p* = 0.5830). Post hoc comparisons using Tukey’s multiple comparisons test showed significant differences between two group pairs (WT Ctrl Dorsal vs CUMS Dorsal, *p* = 0.0078; WT Ctrl Ventral vs CUMS Ventral, p = 0.0469). No significant differences were observed between dorsal and ventral regions within each group. ns, not significant; **p* < 0.05; ***p* < 0.01; *** *p* < 0.001. V3W, vehicle administration of 3 weeks; C1W, corticosterone administration of 1 week; C3W, corticosterone administration of 3 weeks; Ctrl, control; WT, wild type; CUMS, chronic unpredictable mild stress.

**
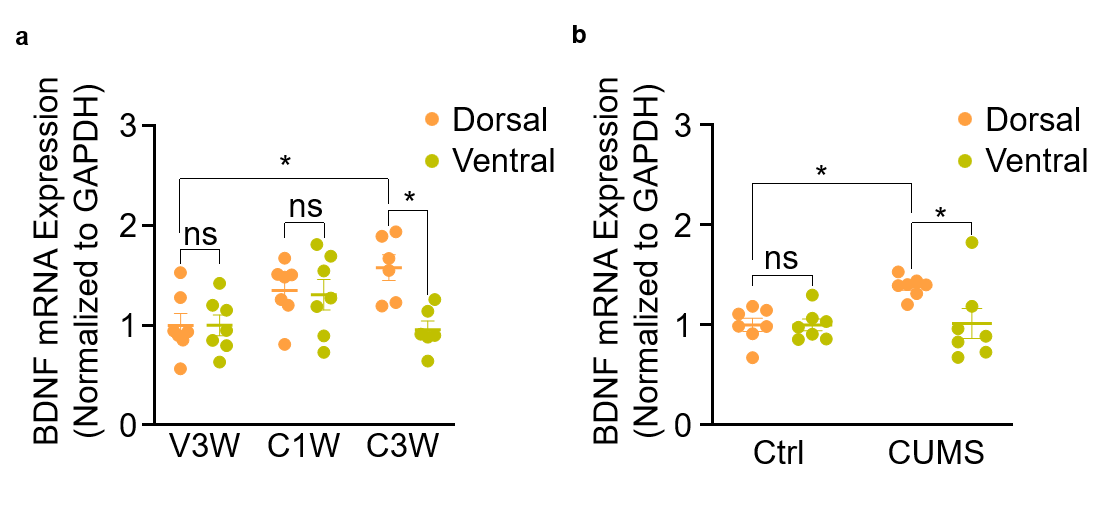
Supplementary Fig. 14 Mouse depression models induce significant upregulation of BDNF in dorsal dentate gyrus.**

**a** BDNF qRT-PCR results in the dorsal and ventral hippocampus of WT mice in a corticosterone induced depression model. Two-way ANOVA revealed a significant main effect of corticosterone treatment (*F*(2, 34) = 4.388, *p* = 0.0202), region (*F*(1, 34) = 0.5159, *p* = 0.0296) and interaction between corticosterone treatment and region (*F*(2, 34) = 4.003, *p* = 0.0275). Post hoc comparisons using Tukey’s multiple comparisons test showed significant differences between several group pairs (WT V3W Dorsal vs C3W Dorsal, *p* = 0.0280; WT C3W Dorsal vs C3W Ventral, *p* = 0.0198). No significant differences were observed between dorsal and ventral regions within V3W and C1W. ns, not significant; **p* < 0.05. **b** BDNF qRT-PCR results in the dorsal and ventral hippocampus of WT mice in CUMS model. Two-way ANOVA revealed significant main effect of CUMS (*F*(1, 24) = 5.003, *p* = 0.0349), region (*F*(1, 24) = 4.342, *p* = 0.0480) and interaction between CUMS and region (*F*(1, 24) = 4.342, *p* = 0.0480). Post hoc comparisons using Tukey’s multiple comparisons test showed significant differences between two group pairs (WT Ctrl Dorsal vs CUMS Dorsal, *p* = 0.0322; WT CUMS Dorsal vs CUMS Ventral, *p* = 0.0415). No significant differences were observed between dorsal and ventral regions within Ctrl. ns, not significant; **p* < 0.05. BDNF, brain-derived neurotrophic factor; V3W, vehicle administration of 3 weeks; C1W, corticosterone administration of 1 week; C3W, corticosterone administration of 3 weeks. Ctrl, control; WT, wild type; CUMS, chronic unpredictable mild stress.
